# Non-canonical Activation of Akt in Serum-Stimulated Fibroblasts, Revealed by Comparative Modeling of Pathway Dynamics

**DOI:** 10.1371/journal.pcbi.1004505

**Published:** 2015-11-10

**Authors:** Tri Hieu Nim, Le Luo, Jacob K. White, Marie-Véronique Clément, Lisa Tucker-Kellogg

**Affiliations:** 1 Computational Systems Biology Programme, Singapore-MIT Alliance, Singapore; 2 Systems Biology Institute (SBI), Clayton, Victoria, Australia; 3 Australian Regenerative Medicine Institute and Faculty of IT, Monash University, Clayton, Victoria, Australia; 4 Department of Biochemistry, Yong Loo Lin School of Medicine, National University of Singapore, Singapore; 5 Department of Electrical Engineering and Computer Science, Massachusetts Institute of Technology, Cambridge, Massachusetts, United States of America; 6 Graduate School of Integrative Sciences and Engineering, National University of Singapore, Singapore; 7 Duke-NUS Graduate Medical School Singapore, National University of Singapore, Singapore; University of Michigan, UNITED STATES

## Abstract

The dynamic behaviors of signaling pathways can provide clues to pathway mechanisms. In cancer cells, excessive phosphorylation and activation of the Akt pathway is responsible for cell survival advantages. In normal cells, serum stimulation causes brief peaks of extremely high Akt phosphorylation before reaching a moderate steady-state. Previous modeling assumed this peak and decline behavior (i.e., “overshoot”) was due to receptor internalization. In this work, we modeled the dynamics of the overshoot as a tool for gaining insight into Akt pathway function. We built an ordinary differential equation (ODE) model describing pathway activation immediately upstream of Akt phosphorylation at Thr^308^ (Aktp^308^). The model was fit to experimental measurements of Aktp^308^, total Akt, and phosphatidylinositol (3,4,5)-trisphosphate (PIP3), from mouse embryonic fibroblasts with serum stimulation. The canonical Akt activation model (the null hypothesis) was unable to recapitulate the observed delay between the peak of PIP3 (at 2 minutes), and the peak of Aktp^308^ (at 30–60 minutes). From this we conclude that the peak and decline behavior of Aktp^308^ is not caused by PIP3 dynamics. Models for alternative hypotheses were constructed by allowing an arbitrary dynamic curve to perturb each of 5 steps of the pathway. All 5 of the alternative models could reproduce the observed delay. To distinguish among the alternatives, simulations suggested which species and timepoints would show strong differences. Time-series experiments with membrane fractionation and PI3K inhibition were performed, and incompatible hypotheses were excluded. We conclude that the peak and decline behavior of Aktp^308^ is caused by a non-canonical effect that retains Akt at the membrane, and not by receptor internalization. Furthermore, we provide a novel spline-based method for simulating the network implications of an unknown effect, and we demonstrate a process of hypothesis management for guiding efficient experiments.

## Author Summary

Influential pathways of cell signalling (such as PI3K/Akt) are routinely communicated using simple textbook-like diagrams that show only the most widely-accepted steps of the pathway. At the same time, there are countless other molecular influences relevant to each pathway, documented in the published literature, and more are being published every week. It should perhaps come as little surprise that during a routine observation of the Akt activation pathway, a simulation of the canonical model was mathematically incompatible with our observed dynamics. To progress beyond the standard, simplified model without testing an unreasonable number of molecular candidates individually, we employed computational modeling to analyze the dynamics of pathway activation. We asked when and where a non-canonical deviation could occur, relative to the canonical pathway. We used the timing of downstream activation to solve for the possible times of upstream initiation. By categorizing unknown effects by their dynamics, we were able to prune away implausible hypotheses using an efficient number of *in vitro* experiments. At the end we had a single plausible explanation for non-canonical Akt activation in our cells, and we confirmed experimentally that Akt is retained at the membrane after PIP3 is no longer present.

## Introduction

Akt, also known as protein kinase B (PKB), promotes cell survival, inhibits apoptosis and contributes to cancer progression, metastasis, and drug resistance [1]. Constitutive activation of Akt is sufficient to cause cancer in mammals [2]. Even subtle or transient effects that increase Akt activation are important to elucidate, because any existing pathways that can heighten Akt activation in normal cells may also be utilized to maintain an excessive activation of Akt in cancer.

Many factors can influence Akt pathway function, but the key steps of Aktp^308^ phosphorylation can be summarized simply. Growth signals and stress pathways activate PI3K (Phosphatidylinositol 3-kinases), which converts PIP2 (Phosphatidylinositol (4,5)-bisphosphate) into PIP3 (Phosphatidylinositol (3,4,5)-trisphosphate). PIP3 is a membrane phospholipid with potent signaling effects, and its concentration is kept low by the PTEN (Phosphatase and tensin homolog) phosphatase, which converts PIP3 back into PIP2 [3]. PIP3 recruits PDK1 (3-phosphoinositide-dependent protein kinase 1 or PDPK1) to the cell membrane [4]. PIP3 also recruits Akt to the cell membrane, where Akt can be phosphorylated at Thr^308^ by PDK1 [5]. Once phosphorylated, Akt dissociates from the membrane and targets many downstream effector substrates [6]. Inactivation of Akt by dephosphorylation is regulated by a variety of phosphatatases, with the PP2A phosphatase known to be particularly important for dephosphorylation of Thr^308^ [7]. We refer to these key steps as **the canonical Akt pathway**. The total net activation of Akt results from multiple competitions between processes of activation and inactivation.

Activation of Akt kinase activity involves phosphorylation of two residues: Thr^308^ in the activation loop and Ser^473^ in the C-terminal hydrophobic motif. The phosphorylation of Akt at Thr^308^ is a prerequisite for full Akt activity, and in some contexts is a better correlate with activity than phosphorylation at Ser^473^[8]. For example, in tumor samples, the phosphorylation of Thr3^08^ is a more reliable biomarker for Akt kinase activity than Ser^473^ [9]. Therefore, we focus on Thr^308^ phosphorylation in this work.

When quiescent cells are stimulated with growth factors, the level of Akt phosphorylation rises from very low to a brief extreme of very high phosphorylation, followed by a decline to a moderate steady level. Indeed many signal transduction pathways have a similar trend (sometimes called “overshoot” or “negative feedback”), which is routinely modeled as an effect of receptor internalization [10,11]. Although many pathways do not display overshoot [12], this peak and decline is a documented feature of Akt dynamics [13,14].

Mathematical modeling with ordinary differential equations (ODEs) is best known for revealing how a set of simple reactions can constitute a complex system with emergent behavior [15,16]. Another use of dynamic models is to identify discrepancies between the accepted mechanisms of a pathway, and the experimental observations of pathway dynamics. For example, we used a dynamic model of pathway activation to uncover unknown effects of a drug regulating apoptosis sensitization [17,18], and to uncover a novel negative feedback loop in TGF-β/Smad signaling [19]. Yet another use of modeling is to study an observed behavior with unknown mechanism [20]. In such cases, computational modeling can illustrate multiple alternative hypotheses, as demonstrated by Hua *et al*. for alternative mechanisms of Bcl-2 action [21], or by Xu et al. for alternative topologies of Erk activation [22]. The peak and decline of Akt phosphorylation is a rich source of indirect information that computational models may be able to exploit.

Previous models of Akt activation dynamics have given limited treatment to the peak and decline behavior. Hatakeyama *et al*. modeled Akt and Erk activation by assuming that receptor internalization would cause Aktp^308^ to decline after its initial peak. Some recent studies [11,23,24] have modeled Akt activation as a part of larger models to study the crosstalk between Akt and other pathways (*e*.*g*. Ras/Raf), and did not investigate the overshoot of Akt. Furthermore, few computational models have incorporated new sources of experimental data about Akt dynamics [25]. We focus on Akt overshoot dynamics for several reasons. Firstly, pathway dynamics can give insight into underlying pathway mechanisms. Secondly, the dynamics of Akt phosphorylation may affect its downstream functional effects. Thirdly, the landmark role of Akt makes it particularly important to understand quantitatively. Countless groups measure phospho-Akt, relying on it to reflect the canonical pathway, including PI3K, PTEN, PIP3, and PDK1 [26].

In this work, we used modeling and experiments to study the peak and decline of Aktp^308^ following the re-introduction of serum in the culture medium of serum-starved fibroblasts. In the first unit of results, we modeled the canonical Akt pathway downstream of PIP3 and upstream of Aktp^308^, comparing model simulations against experimental observations. The canonical model could not explain how the observed PIP3 dynamics would lead to the observed downstream dynamics of Aktp^308^. This major discrepancy led us to reject the canonical model. The second unit of results describes a novel strategy for studying the unknown effect; we generated a series of alternative hypotheses by assuming that a non-canonical effect occurs, with arbitrary dynamics, at any one step of the canonical pathway. For each alternative hypothesis, we found an optimized curve shape for the non-canonical effect, by determining how much deviation would be necessary at each timepoint, in order for the model to fit the data. In the third unit, computational simulations suggested an experiment to differentiate among the alternative hypotheses, and two hypotheses with gross violations of the new experiments were ruled out. In the fourth unit, we predicted, performed, and interpreted another experiment and further narrowed the range of plausible alternatives. This iteration between wetlab and drylab allowed us to conclude that the peak and decline of Akt phosphorylation at Thr^308^ is predominantly caused by a non-canonical effect recruiting or retaining Akt at the cell membrane.

## Results

### Unit One: The canonical pathway cannot explain the delay between PIP3 and pAkt

#### A delay between peak PIP3 activation and peak Akt phosphorylation

**Fig 1:**
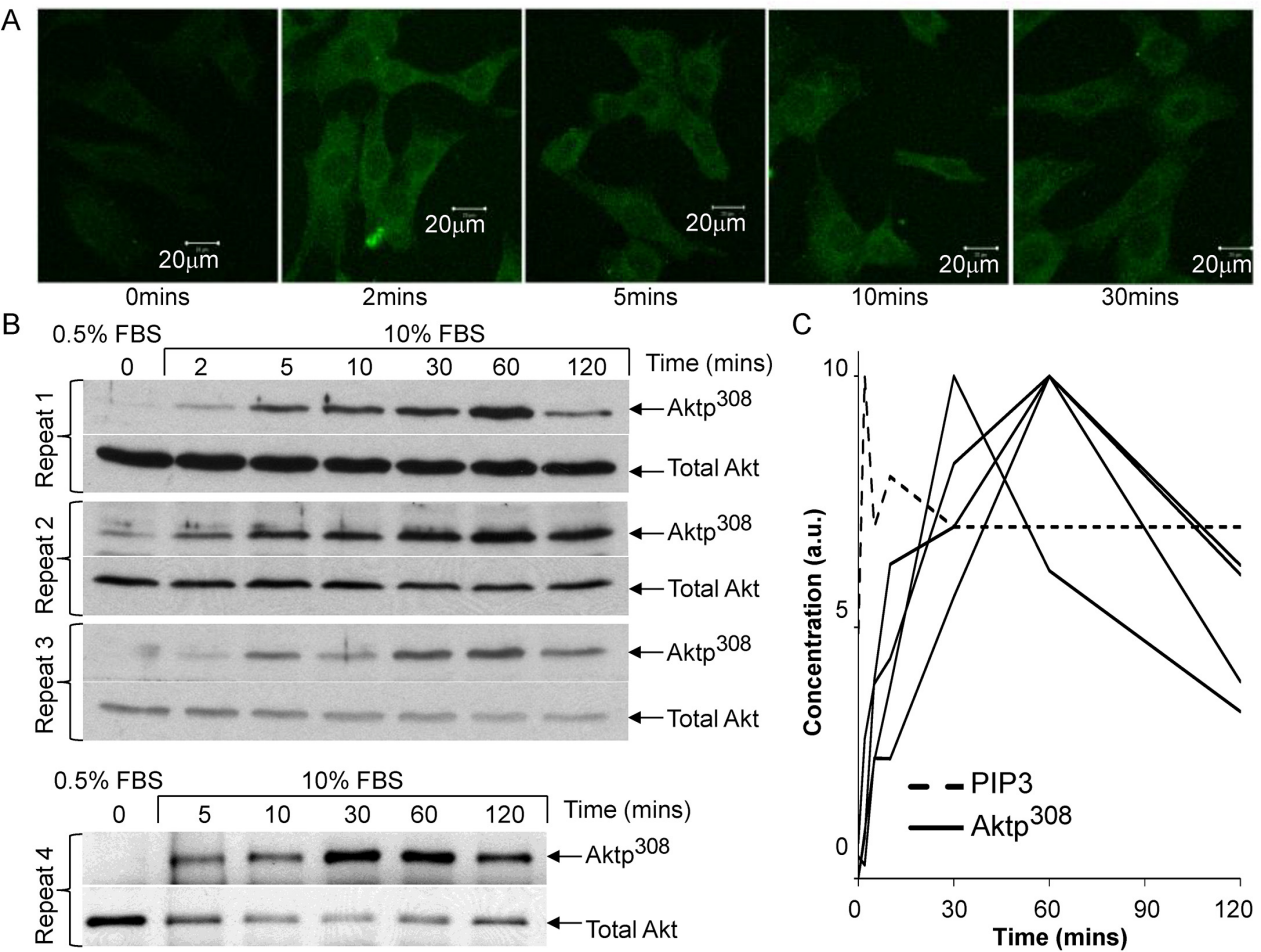
Akt pathway dynamics in serum-stimulated mouse embryonic fibroblasts (MEFs). (**A**) Images of PIP3 immunofluorescence, representative of our replicates, and similar to previously reported dynamics for PIP3 [4,27]. For human readability, micrographs are shown with 5% increased brightness applied to all frames, but the fluorescence intensity was quantified using the unmodified micrographs. (**B**) Immunoblots of Aktp^308^ in serum-stimulated mouse embryonic fibroblasts (MEFs), repeated 4 times. Cells were grown initially in 10% fetal bovine serum (FBS), then incubated 24 hours in culture medium containing 0.5% FBS. Then 10% FBS was re-introduced (“serum stimulation”) and subsequent measurements were taken, relative to the serum stimulation. Time t = 0 indicates cells in 0.5% FBS without serum stimulation. (**C**) Quantified time-series of PIP3 (dotted line) and Aktp^308^ (4 solid lines for 4 experimental replicates) in arbitrary units (a.u.). The densitometry of Aktp^308^ was normalized by the densitometry of total Akt, and then rescaled such that the highest level in each time-series was equal to 10.0 (for consistency with the previous model by Hatakeyama *et al*.) Note that the earliest of the Aktp308 curves peaks at 30 minutes.

As input data for building a model of serum-stimulated Akt phosphorylation, we used previously published time-series measurements of PIP3 abundance and Akt phosphorylation in mouse embryonic fibroblasts (MEFs) [26]. MEFs had been serum-deprived (given 0.5% fetal bovine serum for 24 hours) prior to stimulation at time t = 0 with 10% fetal bovine serum (FBS). In agreement with previous time-series dynamics [4,27], we found that PIP3 peaked at 2 minutes (Fig 1A). Akt phosphorylation at Thr^308^ peaked at 30–60 minutes, before declining to a steady state of activation at 2–4 hr (Fig 1B). Surprisingly, the Aktp308 peak was an order of magnitude later than the PIP3 peak.

Previous models of Akt pathway dynamics produced nearly simultaneous peaks for PIP3 and Akt phosphoryation [10,11], and to the best of our knowledge, previous models of Akt pathway dynamics have not been compared with experimental observations of PIP3 dynamics. The large time difference between PIP3 and Aktp^308^ (Fig 1C) is intriguing considering that PIP3 is not far upstream of Akt phosphorylation in the canonical PIP3/Akt cascade. Recall that PIP3 recruits PDK1 (PIP3-dependent kinase 1), by binding it and recruiting it from the cytosol to the plasma membrane [28]. PIP3 also recruits Akt to the membrane. At the membrane, PDK1 phosphorylates Akt directly at Thr^308^ [5].

Next we asked whether a model simulating these reactions would be capable of creating enough delay after the PIP3 peak to recapitulate the observed dynamics. To be conservative, the model-fitting process focused on explaining the earliest (and least challenging) of the four observed curves, with a peak at 30 minutes.

#### The null hypothesis model

**Fig 2:**
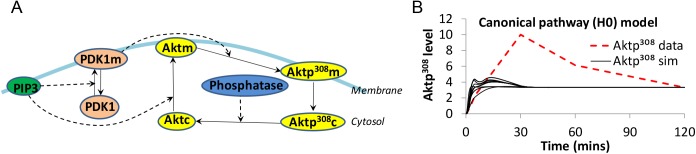
The canonical model of Akt activation. (**A**) Network diagram for the canonical pathway, which is the null hypothesis (H0). The phosphatases capable of dephosphorylating Aktp^308^ are represented by a single entity in the model, named “Phosphatase.” (**B**) Time course simulations of 100 best-fit models for H0 (black solid lines) compared with measured Aktp^308^ time-series (red dashed line) show major, qualitative discrepancies. All simulated time courses were normalized to have the same concentration at t = 120min. See materials and methods for the construction and simulation of the models.

We constructed a model of canonical Akt activation (Fig 2A), starting upstream with PIP3 levels and ending downstream with phosphorylation of Akt at Thr^308^. This model, called H0, functions as our null hypothesis. Some parameters were taken from previously published models [10,11], and others remained to be estimated when fitting the model to observations. The H0 model was constructed using ordinary differential equations (ODEs), where each ODE represents the production and consumption of a molecular species over time. Integrating the ODEs provides a time-evolution (i.e., a simulated trajectory) of the species abundance over time. The reaction equations and parameters of H0 appear in Table 1 and Table 2. This model assumes that Akt dephosphorylation occurs only in the cytosol, based on evidence that the PP2A phosphatase is not present in the membrane fraction of this cell type [29]. PIP3 levels were taken from observations (Fig 1C, dashed line), interpolated into a smooth curve, and plugged into the ODE model [30] for computing the effect of PIP3 on other species in the system. The output of the simulation was Aktp^308^, which consists of both cytosolic Aktp^308^ (“Aktp^308^c”) and membrane Aktp^308^ (“Aktp^308^m”).

**Table 1:** Reactions and rate constants used in the H0 model. The process column describes the reaction equation. The column for reaction velocity indicates the modeled speed of the reaction. Full model ODEs are provided in the materials and methods section.

	Process	Reaction Velocity	Rate constants	Reference
1	PIP3 + PDK1 → PIP3:PDK1m	k1[PIP3][PDK1]	k1 = 1.79	Estimate
2	PIP3:PDK1m → PIP3 + PDK1	k2[PIP3:PDK1m]	k2 = 9.9	Estimate
3	PIP3 + Aktc → PIP3:Aktm	k3[PIP3][Aktc]	k3 = 5.27	Estimate
4	PIP3:Aktm → PIP3 + Aktp^308^m	k4[PIP3:PDK1m][PIP3:Atkm]/	k4 = 2450	[31]
		(k5 + [PIP3:Aktm])	k5 = 68000	[31]
5	Aktp^308^m → Aktp^308^c	k6[Aktp^308^m]	k6 = 0.49	Estimate
6	Aktp^308^c → Aktc	k7[Phosphatase][Aktp^308^c]/	k7 = 5	Estimate
		(k8 + [Aktp^308^c])	k8 = 1.65	Estimate

**Table 2:** Initial concentrations of the species in the H0 model. Note that the PIP3 time-course is interpolated from experimental observations and not governed by the equations of the model. Aktp^308^ is defined to be the sum of Aktp^308^m and Aktp^308^c. Initial concentrations of PIP3:PDK1m and Aktp^308^c were chosen such that the total (membrane plus cytosolic) amount of PDK1 (PDK_total) and Akt (Akt_total) would be 10.

Species	Initial concentration
PIP3	from measured data (linear piecewise spline)
PDK1_total	10
PDK1	9.75
PIP3:PDK1m	[PPDK1_total]—[PDK1]
Akt_total	10
Aktc	9.988
PIP3:Aktm	0.001
Aktp^308^m	0.001
Aktp^308^c	[Akt_total]—[Aktc]—[PIP3:Aktm]—[Aktp^308^m]
Aktp^308^	[Aktp^308^m] + [Aktp^308^c]
Phosphatase	0.24

Parameterizing the model included several assumptions. The parameters were constrained to produce a steady state for the initial concentrations of the system at time t = 0. The binding rate of PIP3:PDK1m to PIP3:Aktm was derived from [10,31]. The PIP3 rate constant for binding to Aktc was allowed to differ from the PIP3 rate constant for binding PDK1. Finally, based on the observation that PDK1 and Akt have undetectable levels at the membrane in serum-deprived cells [26], we required that the initial state of the system must have all PDK1 and Akt in the cytosol. Tables 1 and 2 list the rate parameters and initial concentrations that were determined by parameter estimation. The parameter estimation process (see Materials and Methods) maximized the agreement between model simulations and experimental observations (Fig 1C).

#### Rejection of the null hypothesis

Because there are unknown parameters in the H0 model, we considered a family of different models, each containing the same set of ODEs as in Table 1, but with different values for the variable parameters. Each model in the H0 family may contain independently optimized rate parameters. Extensive efforts to fit the H0 model family to the observed Aktp^308^ measurements were unsuccessful, including large numbers of random restarts with global and local optimization methods. The assortment of simulated trajectories contains no models with a 30 minute peak followed by a decline. The assortment of simulated trajectories superimposed with measurements of Aktp^308^ shows no good match (Fig 2B). Some parameters were found that allowed some models to display a peak and decline in Aktp^308^, but these were early peaks, mirroring the 2 minute peak of PIP3. The failure to match the model to the data suggests that no instance of the H0 model family is capable of recapitulating the observed dynamics of Aktp^308^ with the given PIP3 data as input. In other words, it is difficult to explain the dynamics of Aktp^308^ based only on the canonical PIP3 dynamic at the membrane.

### Unit Two: Generating alternative hypotheses

There is published evidence for many other mechanisms to influence Akt. Akt activation can be affected by Tcl1 [32], ILK [33], PIKE [34], and PAK1 [35]; by chemical modifications such as ubiquitylation [36], phosphorylation by CK2 [37], phosphorylation by Ack1 [38], disulfide formation [39] and O-GlcNAcylation [40]; and by regulatory influences such as lipid rafts [41], reactive oxygen species [42], SHPS-1 [43], TTC3 [44], CTMP [45], and ATP-binding [46]. In addition, Akt binds over 100 known target substrates [47], any of which could exert a localization or scaffolding effect. Another list could be made for regulators of PDK1 or PP2A. From the vast array of non-canonical mechanisms that could affect Akt phosphorylation, we do not know which are applicable to our context; many of these effects have been identified in cancer cells with mutated Akt behavior, or in specialized cell types, or under extreme conditions. Rather than designing an alternative hypothesis for each published biochemical effect, we instead ***categorized the universe of possible non-canonical effects according to which step of the canonical pathway is affected*.** That is, we established one alternative hypothesis for each step of the canonical pathway. We refer to this approach as a top-down strategy for coverage of alternative hypotheses. Categorizing hypotheses by their “point of intervention” will allow us to accept or reject hypotheses depending on the dynamics of the effect.

Starting with six steps of the pathway corresponding to six alternative hypotheses, we immediately discarded the hypothesis of perturbed PDK1 recruitment to the membrane, based on our time-series measurements of membrane PDK1 [29], and repeated in S1 Fig. PDK1 recruitment dynamics were a close mirror of the PIP3 dynamics, indicating no non-canonical effect perturbing or delaying the PDK1 dynamics.

**Fig 3:**
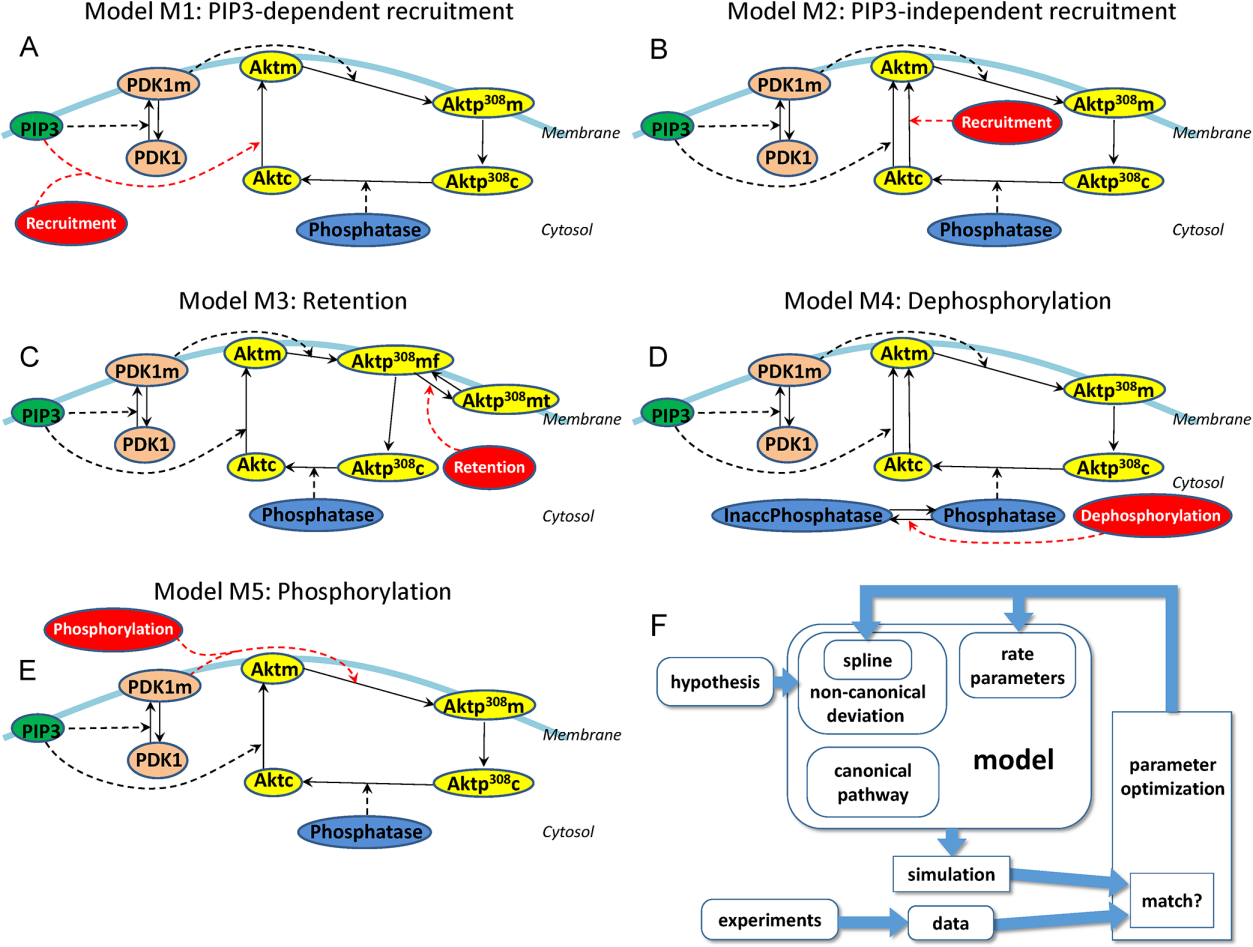
Five alternative hypotheses, each with one non-canonical effect (in red). Enumerating the steps of the canonical pathway, downstream of PIP3 and upstream of Aktp^308^, yielded five alternatives: (**A**) the PIP3-dependent recruitment model M1, (**B**) the PIP3-independent recruitment model M2, (**C**) the retention model M3, (**D**) the dephosphorylation model M4, and (**E**) the phosphorylation model M5. For each alternative model, a pseudo-reaction (shown in red) perturbs the canonical pathway, and the strength of the deviation over time is determined by a time-dependent spline curve (not shown). In the retention model (M3), the membrane-bound phosphorylated Akt (Aktp^308^m) is divided into two hypothetical subpopulations: membrane-free (Aktp^308^mf, which can dissociate from the membrane) and membrane-trapped (Aktp^308^mt, which cannot dissociate from the membrane). In the dephosphorylation model (M4), cytosolic phosphatases (including PP2A and other factors capable of dephosphorylating Aktp^308^) are represented by two subpopulations: normal phosphatases (Phosphatase), and inaccessible/inactive phosphatases (InaccPhosphatase). **(F)** Flowchart for optimizing each alternative model, with its non-canonical effect, to fit the experimental data. The model consists of a canonical network with rate parameters, and a non-canonical deviation. The non-canonical deviation was encoded as a pseudo-species with an unknown spline curve for its time series profile. Unknown parameters of the model (including the knots of the spline curve and some of the biochemical reaction rates) were optimized, with many cycles of iteration, until the simulated model was able to fit the data.

For the remaining 5 alternative hypotheses S2 Fig), we do not know the molecular mechanism that would cause each hypothetical deviation, so we cannot model the alternatives with explicit enzyme kinetics. Instead, we constructed an arbitrary spline curve to represent the magnitude of the hypothetical deviation over time. The shape of the spline curve can later be optimized for the model to fit the observed trends (see “Using splines to represent non-canonical deviations” and subsequent sections in the Methods section). In other words, optimizing the perturbed model to fit the data would solve for the shape of the spline curve, which tells us when the deviation would occur. Each spline curve was integrated into the canonical model by adding a pseudo-reaction (Fig 3) at the appropriate step of the pathway. The pseudo-reaction should not be misinterpreted as representing a true biochemical mechanism; it is only an interface between the spline curve and the differential equations of the network model.

Using splines and pseudo-reactions as described, we converted the five alternative hypothesis into five computational models (Fig 3): Akt recruitment could be altered through a PI3K/PIP3-dependent mechanism (“PIP3-dependent recruitment model”, M1, Fig 3A), Akt recruitment could be altered by a mechanism that is independent of PI3K/PIP3 (“PIP3-independent recruitment model”, M2, Fig 3B), Akt translocation from membrane back to cytosol could be blocked or delayed (“retention model”, M3, Fig 3C), or Akt dephosphorylation could be disrupted (“dephosphorylation model”, M4, Fig 3D), or the phosphorylation of membrane-localized Akt could be altered (“phosphorylation model”, M5, Fig 3E). Fig 3F shows a flowchart for how each model, with its non-canonical effect, was fit against the data (see also the “parameter estimation” sections of Methods).

Our approach to managing the alternative hypotheses was first to study how the hypotheses could be differentiated from each other. This led to a small number of follow-up experiments. After each experiment, any hypothesis that grossly violated the observed trends was ruled out.

### Unit Three: Differentiating among the alternative hypotheses by measuring localization

#### Alternative models predict different dynamics of membrane fractions

**Fig 4:**
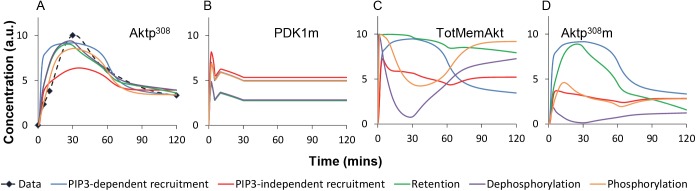
Time course simulations of the five alternative models. (**A**) Aktp^308^, (**B**) membrane PDK1, (**C**) membrane-bound total Akt (TotMemAkt), and (**D**) membrane-bound phosphorylated Akt (Aktp^308^m) from the five alternative models. Model predictions suggested that measuring TotMemAkt and Aktp308m could discriminate between alternative hypotheses. TotMemAkt is equal to Aktp^308^m + Aktm (all membrane-bound Akt, both phosphorylated and unphosphorylated). Aktp^308^ is equal to Aktp^308^m + Aktp^308^c (the total amount of Aktp^308^ both at the membrane and in the cytosol). For the retention model, Aktp^308^m is equal to Aktp^308^mf + Aktp^308^mt.

All five models were successfully fit (Fig 3F and “parameter estimation” in the Methods) to achieve agreement with the dynamics of Aktp^308^ (Fig 4A). This indicates that additional data will be necessary for differentiating among the alternatives. Simulations of membrane-bound PDK1 (Fig 4B) showed no major differences between the alternative models, suggesting that use of this available PDK1 data would not distinguish correct from incorrect models. In contrast, simulation of the five alternative models revealed qualitative differences in the dynamics of total membrane-bound Akt (TotMemAkt, Fig 4C) and total membrane-bound phosphorylated Akt (Aktp^308^m, Fig 4D). Multiple parameterizations of each model showed similar effects, motivating us to perform fractionation measurements of Akt.

#### Measurements of the membrane fractions

**Fig 5:**
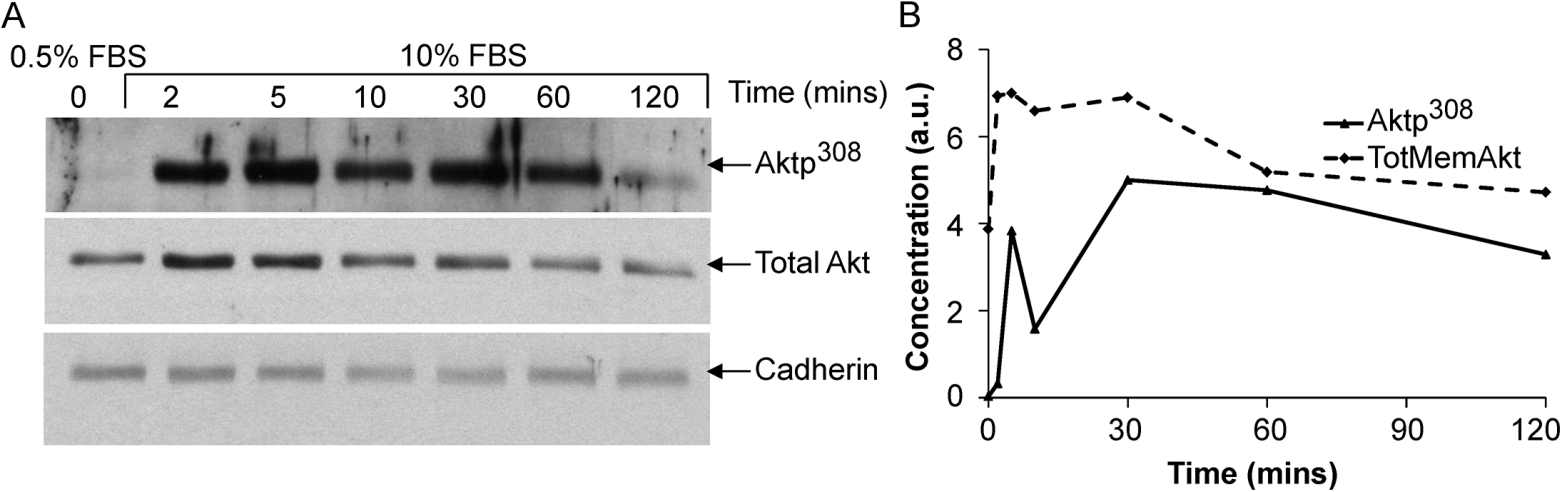
Membrane fractionation experiment. **(A)** Representative immunoblot blot of the membrane fraction of the cell lysate, probed for phosphorylated Akt (Aktp^308^m, with n = 3 repeats), and total Akt (TotMemAkt, with n = 6 repeats). **(B)** The median relative increment of Aktp308m and TotMemAkt were plotted according to the methods and tables in S1B Dataset. Briefly, the quantified band intensities were normalized by Cadherin, and then plotted as median increments.

Fractionation experiments were performed to obtain time-series measurements of membrane-bound phosphorylated Akt (Aktp^308^m) and total membrane-bound Akt (TotMemAkt = Aktp^308^m + Aktm). Fig 5A shows a representative blot, and the complete set of n = 3 or n = 6 replicates is shown in S1A Dataset. Considerable noise is present, typical for this type of experiment, but there is a consistent trend of Aktp^308^m and TotMemAkt remaining localized at the membrane for a prolonged period of time after the 2–5 min peak of PIP3. Fig 5B plots our calculation of the median effect, as described in the caption. TotMemAkt levels (diamonds with a dashed curve) peaked at 5 minutes and stayed high for 30 minutes before decreasing slightly. Aktp^308^m levels (triangles with solid curve) increased sharply and remained high for at least 30 minutes, although some replicates showed a dip at 10 minutes. As described previously, PDK1m levels (S1 Fig) peaked at 2 minutes and quickly decreased.

#### Fitting the alternative models to the fractionation time-series

**Fig 6:**
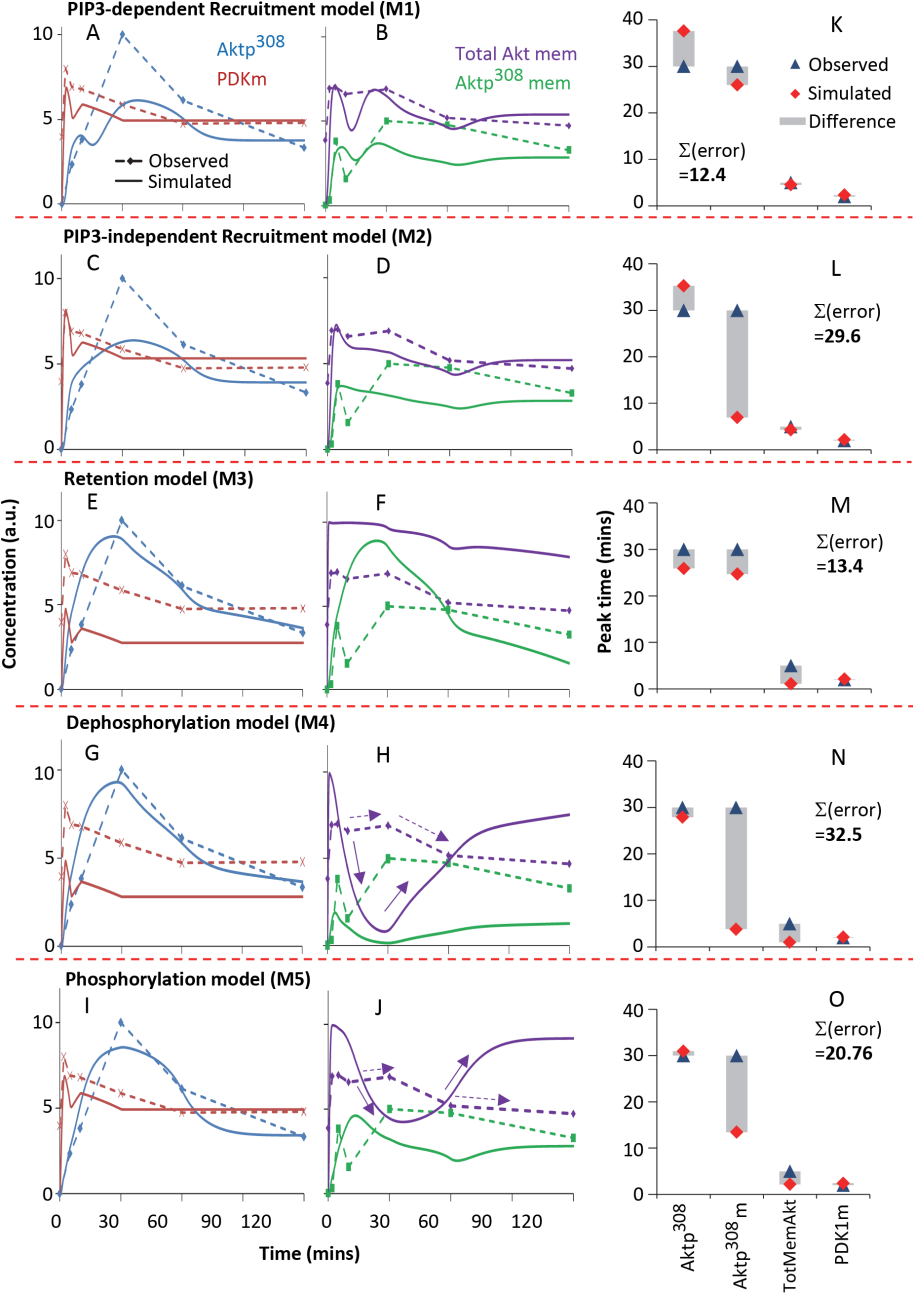
The fits of alternative models to the experimental data. Comparisons show experimental time-series (dotted lines) and simulated time courses (solid lines) for (**A-B**) the PIP3-independent recruitment model, (**C-D**) the PIP3-dependent recruitment model, (**E-F**) the retention model, (**G-H**) the dephosphorylation model and (**I-J**) the phosphorylation model, each with Aktp^308^ in the total cell lysate (blue curve), membrane PDK1 (red curve), TotMemAkt (purple curve), and membrane Aktp^308^ (green curve). All plots of concentration (abundance) use arbitrary units. The criteria for match are whether the model can reproduce the peak time, peak duration and peak amplitude of the observed trend. The purple curves in (H) and (J) were considered implausible by human inspection and their scores showed high violations (S1 Tables) due to the mismatch shown by the purple arrows. The green curves in (**F**), (**H**) and (**J**) were considered problematic but not necessarily in violation of the data. (**K-O**) The peak time of each model (red diamond) was compared with the observed peak (blue triangle), and gray bars indicate the difference. Σ(err) shows the sum of the error in peak time, across the four species of comparison.

Using the observed PIP3 levels as an input to the system, we then had 4 time-series curves available for the model-fitting process: Aktp^308^m (green, membrane-localized phospho-Akt), Aktp^308^ (blue, total phospho-Akt regardless of localization), TotMemAkt (purple, total membrane-localized Akt, both unphosphorylated and phosphorylated), and PDK1m (red, membrane-localized PDK1). After each model was fit to the data, a simulated time-course of the system was computed and compared against the time-series data including total cell lysate Aktp^308^ (Fig 6, blue curves) and membrane fraction experiments (Fig 6, red, green and purple curves). Because the experiments measured relative fold-change rather than absolute concentration, we measured the quality of model fit based on properties of the Akt peak: peak time, peak duration (width) and peak amplitude. Note the temporal properties of the peaks are not affected by relative scaling on the measurements, which is important because Western blot experiments give better accuracy for relative fold-change than for absolute concentration. Quantified scores are provided in S1 Tables. We also plot the comparisons in Fig 6 for visualization.

The **PIP3-dependent recruitment model, M1** (Fig 6A and 6B) exhibited reasonable agreement with the observed peaks in all four measured time-series. This model in fact had two peaks, the first due to the PIP3 peak, and the second due to the non-canonical deviation, but only the main peak contributed to the quantitative scoring (peak time, peak width, peak amplitude, in S1 Tables). The **PIP3-independent recruitment model, M2** also showed reasonable agreement with the four time-series trends (Fig 6C and 6D), but its similarity to the green Aktp^308^m curve was mediocre: the model had an early peak and plateau for Aktp^308^m, while the measurements showed a later peak. This difference caused M2 to have a poor score. The **retention model, M3** (Fig 6E and 6F) agreed well with the pAkt dynamics (blue) and had a good score for the fit of its peaks to the experimental peaks. By eye, one can see that M3 has weak similarity with the early timepoints of the (green) Aktp^308^m time-series, but the scoring of peak time/width/amplitude does not penalize the lack of secondary peak at 5min. Other weaknesses were the fold-change of the TotMemAkt (purple), and a discrepancy in the levels of cytosolic Aktp^308^ from 2–30 minutes (S3 Fig). Cytosolic Aktp^308^ was a noisy measurement that we have not emphasized, but cytosolic Aktp^308^ was not zero, while the retention model had very little cytosolic Aktp^308^ from 2–30 minutes. In sum, the retention model had several weaknesses, but no gross violation of the experimental trends, given the level of experimental noise, and it had a good numerical score for the fit of its peaks. The **dephosphorylation model, M4** (Fig 6G and 6H) had a bad score due to gross disagreement for Aktp^308^m (green) and TotMemAkt (purple) at 30 minutes. The model dipped near zero for both membrane species at 30 minutes, while the experimental observations showed peaks at 30 minutes. This difference is irreconcilable. Finally, the **phosphorylation model, M5** agreed with the data in Fig 6I, but it had weak resemblance to the dynamics of Aktp^308^m (Fig 6J, green curves), and outright violation of the dynamics of TotMemAkt (Fig 6J, purple curves). Note that the phosphorylation model and dephosphorylation model exhibited similar dynamic trends, and both violated the new experimental observations. To display how well the 5 alternative models recapitulated the observed peaks for each species, we plotted the observed and simulated peaks, with grey bars between them to highlight discrepancies (Fig 6K–6O). The PIP3-dependent recruitment model (M1) and the retention model (M3) accurately recapitulated when the four species would peak, but the PIP3-independent recruitment model (M2) and dephosphorylation model (M4) had significant errors in the peak time for Aktp^308^m. In sum, we have identified multiple gross violations in the dephosphorylation model (M4) and the phosphorylation model (M5), and possible weaknesses in all other models except M1.

### Unit Four: Differentiating among the alternative hypotheses by blocking the upstream input

#### Simulating the suppression of PIP3 levels

**Fig 7:**
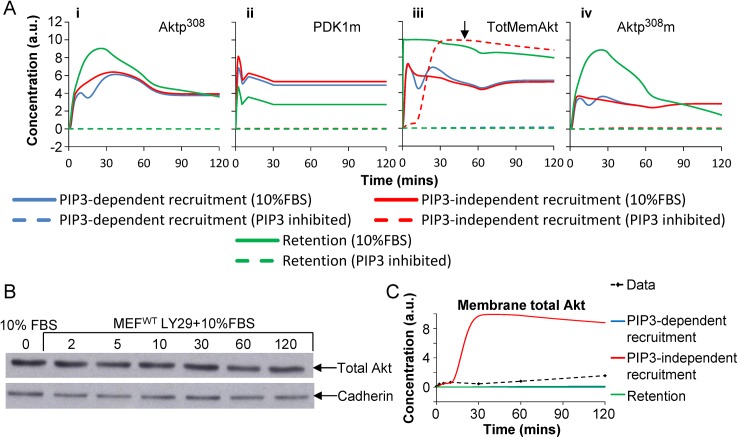
Model predictions and experimental measurements of total membrane-bound Akt after LY29 treatment. (**A**) Time course simulation of: (**i**) Aktp^308^, (**ii**) membrane PDK1, (**iii**) total membrane-bound Akt, and (**iv**) membrane phosphorylated Akt. Simulations used three alternative models: PIP3-dependent recruitment, PIP3-independent recruitment, and retention. The simulations of the “control” experiments (solid lines, called 10%FBS) are repeated from Fig 6 and they used the experimentally observed PIP3 levels as upstream input. The simulations of PIP3 inhibition (dashed lines) used the same models and same parameters, but the PIP3 input curve was set to be constant, at the observed level of PIP3 in unstimulated cells. Model predictions suggested that total membrane-bound Akt (black arrow) has non-trivial dynamics under the PIP3-independent recruitment hypothesis. (**B**) Immunoblot of membrane fraction Akt in serum-stimulated MEFs after pre-treatment with LY29 (representative of n = 3 repeats, other repeats not shown). (**C)** Quantified measurements of TotMemAkt after LY29 treatment (dashed black line) were plotted for comparison with simulations of the three alternative models (solid color lines). Black dashed line: observed time-series for TotMemAkt (n = 3 replicates). Solid color lines: simulated time course of membrane total Akt from each model.

Three models remained under consideration: PIP3-dependent recruitment, PIP3-independent recruitment, and retention. We simulated serum-induced effects the three models while suppressing the PIP3 levels (the canonical input). The serum-induced deviation (the non-canonical input) was maintained. As expected, output was suppressed in all three models (Aktp^308^ in Fig 7A(i), dashed lines; Aktp^308^m in Fig 7A(iv), dashed lines). There was a significant difference among the models for the predicted levels of total Akt at the membrane (Fig 7A(iii), dashed lines). Akt would remain cytosolic for the retention model and PIP3-dependent model, but Akt would accumulate at the membrane under the PIP3-independent recruitment model.

#### LY29 experiments rule out PI3K-independent recruit

Based on the simulations of Fig 7A, we proposed to measure the dynamics of TotMemAkt, after serum stimulation, with and without suppression of PIP3. Lipids are difficult to target directly, so we treated cells with LY294002 (LY29), a small-molecule inhibitor of PI3K. (Recall that PI3K is immediately upstream of PIP3.) LY29 treatment is not necessarily identical to PIP3 suppression, but if LY29 treatment would fail to suppress serum-stimulated recruitment of (unphosphorylated) Akt to the membrane, that would still be compelling support for a model with PIP3-independent recruitment (not to mention PI3K-independent recruitment).

**Fig 8:**
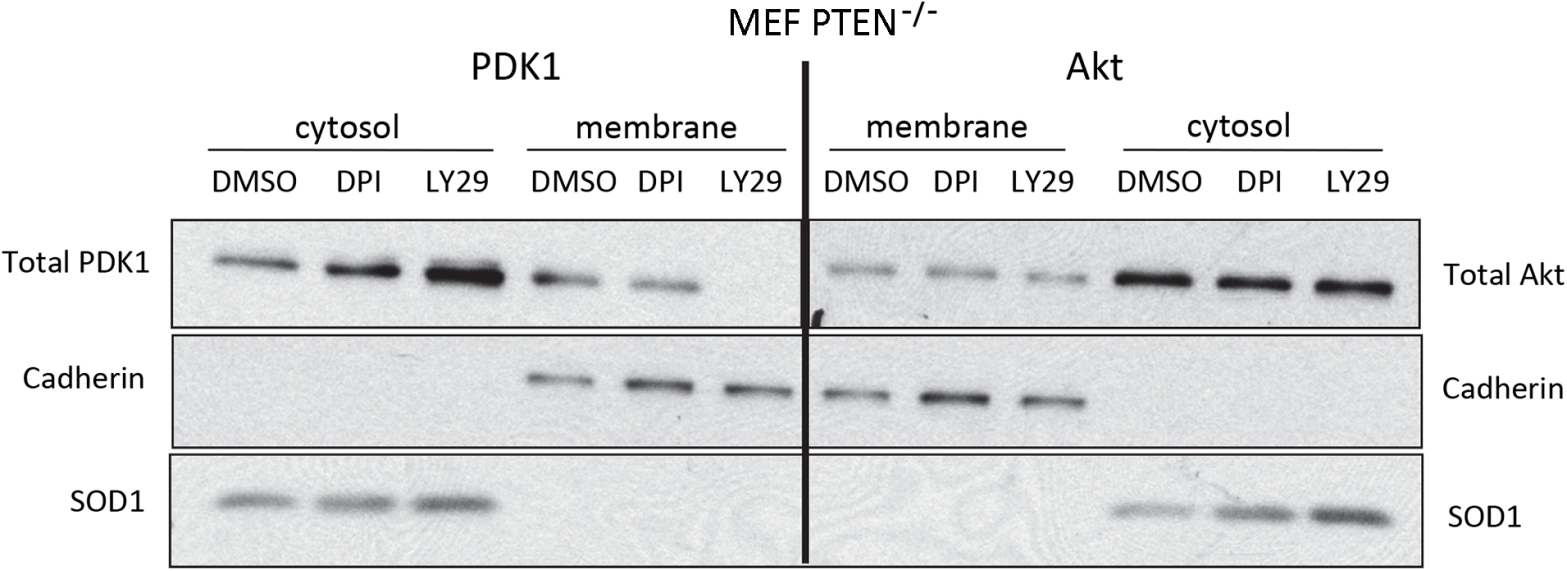
Akt is retained at the cell membrane after a decrease in PIP3. MEF PTEN-/- cells were treated with 25μM LY29 or 6μM DPI for 2hrs before the cytosol and membrane fractions were separated. Abundance of total Akt or total PDK1 were assessed in each fraction by Western blot. The fractionation of membrane-bound from cytosolic is demonstrated by the presence of Cadherin and absence of SOD1 (superoxide dismutase-1). Treatment with DPI (diphenylene iodonium) can be viewed as providing an additional control for the effect of LY29 toward the membrane-bound pool of Akt, because we had previously shown DPI treatment affects phosphorylation of the cytosolic fraction of Akt but not the membrane-bound fraction [48]. DMSO: dimethylsulfoxide.

The membrane fraction of total Akt (TotMemAkt) was measured in serum-stimulated fibroblasts with and without LY29 pre-treatment (Fig 7B). The levels of TotMemAkt were roughly flat, with no non-canonical boost. Fig 8B shows a quantified version of the observed trend (black dashed line), superimposed with three colored curves for the three alternative models. LY29 blocked the non-canonical effect, indicating that LY29 and PI3K are upstream of the non-canonical effect. Combined with the earlier weaknesses in the PIP3-independent recruitment model and the current failure, we ruled out the PIP3-independent recruitment model (red curve), and we retained the models with PIP3-dependent recruitment or retention.

Finally, to differentiate between the last two plausible models, we must study the dynamics of the pathway turning off, rather than the dynamics of turning on. Serum stimulation can create a high level of transient activation, but after a few minutes, the upstream factors (PIP3 and PDK1) reach a moderate steady state that is neither fully active nor fully inactive. To observe the dynamics of shutting the system off, we must start the system in a fully activated state, constitutively active, not partly activated or transiently activated by serum. Therefore to achieve constitutively high levels of PIP3 in resting cells, we used homozygous deletion of PTEN (with no change to other lipid phosphatases that consume PIP3.) We then treated with the LY29 inhibitor to observe the pathway shut down. After 2 hours, PDK1 was absent from the membrane, indicating that the pathway upstream of PDK1 had shut down (Fig 8). However, Akt was still present at the membrane after 2 hours, indicating that Atk was retained in the absence of its canonical upstream activator. This experiment also includes cell treatment with DPI (diphenylene iodonium), which is explained in the figure caption. We conclude that model M3 is correct, and that non-canonical retention of Akt occurs in mouse embryonic fibroblasts.

## Discussion

Aktp308 phosphorylation clearly exhibits overshoot behavior, but the time-scale of the overshoot was considerably later for Aktp308 than for PIP3 (Fig 1). In the first unit of our results, the canonical model was unable to explain the delay between the peaks of PIP3 and pAkt (Fig 2), suggesting that some non-canonical effect causes the overshoot dynamics of Aktp308. A critical consideration for the interpretation of Fig 2 is parameter estimation, which is never guaranteed to find globally optimal values. If our estimated parameters are significantly worse than optimal, then a correct hypothesis could appear to violate the observations. In other words, our search is incomplete. Incompleteness of search is an obstacle to claiming that a model can’t fit a dataset. This is relevant when ruling out the canonical model, and also when claiming that altenative models can’t satisfy the data. To address this risk, we pursued multiple complementary methods of parameter estimation and a large number of random restarts before accepting a high-violation model to be “best-fit” to the data. Secondly, we avoided ruling out models unless they showed gross violations of the observed trends. Third, we could sometimes find an underlying reason or explanatory intuition for why the failure would be unavoidable. Future work could improve our confidence, with less need for human judgment, when testing whether any parameters exist for a given model to match a given behavior.

After finding the canonical model to be inadequate, we adopted a novel top-down strategy for exploring alternative hypotheses: we assumed there was a single non-canonical effect responsible for the majority of the deviation, and we systematically enumerated the steps of the canonical pathway as the possible places where the deviation could occur (Fig 3). Furthermore we developed a novel spline-based method for explicitly representing the time-course of an unknown non-canonical deviation. We inserted non-canonical deviations, one for each step of the pathway, to create a set of alternative models. Each non-canonical devation is encoded using a pseudo-reaction driven by a pseudo-species with unknown time-course. The model (with unknown time-course for the pseudo-species) was optimized for best match with the data. In essence this approach carries out the following assumption: the observed Akt curve(s) should be equal to the network-propagated effects of PIP3, plus the network-propagated effects of the non-canonical deviation. $\mathrm{NetworkPropagate}\left(\vec{PIP3}\right)+\mathrm{NetworkPropagate}\left(devia\vec{tio}n\right)=\vec{pAkt}.$ Because we knew the pAkt curve and the PIP3 curve, we attempted to solve for the curve of the unknown deviation, which means solving for the values of the spline knots. Values for the spline knots were obtained using stochastic global optimization methods for parameter estimation of the model. Parameter estimation adjusted the values of the spline knots and all other variable parameters, many times in iteration (Fig 3F), to maximize agreement with the observations. The use of an explicit curve to encapsulate the unknown deviation provided a complete model of the perturbed system, capable of being simulated (Box 1) and scored (S1 Tables). Simulations could then illustrate possible implications and side-effects of each alternative hypothesis.

When a model does fit experimental data, we should not over-interpret the model as describing how the alternative hypothesis would necessarily work. It is merely an illustration of how it ***might*** work. This distinction arises because the parameter estimation process may encounter multiple minima, while having no fundamental basis for preferring one minimum over another. The global minimum is not necessarily the ground truth (even if the ground truth were a single entity, and even if we could identify the global minimum). Fitting single models allows us to conclude simply that there exists at least one parameterization allowing this hypothesis to fit the data. Fitting a family of models with incrementally different parameterizations is potentially more powerful, but in preliminary trials it yielded results similar to single-model fitting (S1 Document). For future work, when more datapoints are available so the variance can be estimated, we hope to rank alternative models by quality of fit to the entire experimental distribution, similar to the work of Vyshemirsky et al. with Bayesian ranking of models [49] or the work of Eydgahi et al. with Bayesian scoring of parameterizations [50].

**Box 1. Pseudo-code for Simulating an Alternative Model**

1. Encode the hypothetical effect with linear spline ***v***:
  1.1 Find the ODEs that are affected by the hypothetical effects
  1.2 Introduce ***v*** to the canonical ODE model based on the corresponding hypothesis
    (see Eqs 10–20 in Materials and Methods)
  1.3 Let ***v*** (*t*) be the value of ***v*** at time *t* minutes.
    1.3.1 The spline knots ***v***(0), ***v***(2), ***v***(5), ***v***(10), ***v***(30), ***v***(60), ***v***(120) are model parameters
    1.3.2 for time *t* between knots ***v***(*t*_1_) and ***v***(*t*_2_)
***v***(*t*) = ***v***(*t*_1_) + (***v***(*t*_2_)-***v***(*t*_1_))*(*t*-*t*_1_)/(*t*_2_-*t*_1_)
2. Simulate the ODE model:
  2.1 For *t* = 0 to 120 minutes
    2.1.1 For each model input species (*e*.*g*. PIP3) ***i***
***i***(*t*) is a spline-fitted value of ***i*** at time *t* (no simulation)
    2.1.2 For each hypothetical effect species ***v***
***v***(*t*) is a spline-fitted value of ***v*** at time *t* (no simulation)
    2.1.3 For each remaining species ***x***
***x***(*t*) is calculated by integrating the system ODE at time *t*,
      given the values ***i***(*t*) and **v**(*t*) having been calculated in 2.1.1 and 2.1.2

To distinguish among alternative hypotheses, two sets of follow-up experiments were pursued. In unit 3 of the results, membrane fraction time-series excluded the phosphorylation model and the dephosphorylation model because they couldn’t explain why Akt would remain localized at the membrane after PIP3 levels peak and decline. In unit 4, LY29 time-series experiments showed that the non-canonical effect is dependent on PI3K (Fig 7), leaving little possibility for PIP3-independent recruitment to be correct. Finally we applied LY29 to constitutively activated cells and found that the non-canonical presence of Akt at the membrane remained detectable even when PIP3 was no longer sufficient to recruit PDK1 to the membrane (Fig 8). Although recruitment and retention may co-exist in some equilibrium, this experiment shows that retention is more accurate than recruitment to characterize the effect. Table 3 summarizes which models were considered viable before and after each stage.

**Table 3:** Summary of outcomes for each model in the top-down strategy. The data from four experimental tests were applied to the null hypothesis (H0) and five alternative hypotheses.

		Test			
		Aktp^308^ time-series	Membrane time-series	LY29 time-series	Constitutive Activity + LY29
Model	Canonical Model (H0)	Fail	*prior fail*	*prior fail*	*prior fail*
	PIP3-dependent recruitment (M1)	**Pass**	**Pass**	**Pass**	Fail
	PIP3-independent recruitment (M2)	**Pass**	**Pass**	Fail	*prior fail*
	Retention (M3)	**Pass**	**Pass**	**Pass**	**Pass**
	Dephosphyrolation (M4)	**Pass**	Fail	*prior fail*	*prior fail*
	Phosphorylation (M5)	**Pass**	Fail	*prior fail*	*prior fail*

Many common experimental methods measure only relative fold-change and not absolute abundance. Furthermore, measurements contain many sources of error, which do not necessarily follow normal distributions. For example, immuno-blots generally deviate farther from an ideal linear response when the protein has very low abundance or very high abundance. The standard deviation of the mean is a useful measure of error, but is potentially misleading in time-series experiments because there is greater consistency in the timepoint-to-timepoint trends for each batch, than there is from batch to batch in replication of the time-series experiment. There are statistical approaches for analysis of grouped measurements, but such approaches are problematic when combined with other sources of error. Instead we reduced the observed datasets to a low-resolution representation of curve shape, using three types of information: the time of peak abundance, the width of the peak, and the fold-change from peak to steady-state. For plotting, we used the median rather than the mean (Fig 5) and to communicate the spread of the data, we have published primary data from all replicates. Each step is rigorous but we refer to the process as semi-qualitative because the curve-shape features have lower resolution (fewer parameters) than the original dataset, and because the decision which alternative hypotheses to reject included human inspection of outliers, rather than pure automation. However, the steps could be performed using greater automation. The curve-shape scoring is automated, and deciding which models are in gross violation of the curve shape scoring could be performed by thresholding.

Several aspects of this study may interest Akt specialists. One contribution of this work is analyzing the temporal offset between PIP3 dynamics and Akt dynamics, although the existence of this offset is not novel [4,6,26,51]. Our more important contribution for Akt biology is finding that Akt is retained and the membrane after its upstream activators decline. The following are some known non-canonical effects that might mediate Akt retention at the membrane: (1) Akt can bind PI34P (phosphatidylinositol(3,4)-bisphosphate) in addition to PIP3 [52], and so PI34P might be responsible for the prolonged localization of Akt at the membrane after PIP3 levels decline. If PI34P levels are dependent on dephosphorylation of PIP3, then PI34P levels would also be blocked by LY29, satisfying the criteria of PI3K-dependence. (2) Pak1 can serve as an anchor for localizing Akt at the plasma membrane [35]. The Pak1 effect would likely be PI3K-dependent because PI3K activates Rac1, which activates Pak1. (3) NHE1 is an integral membrane protein that promotes Akt signaling through an indirect scaffolding effect [53]. The antiporter activity of NHE1 is known to be PI3K-dependent [54], suggesting that its Akt scaffolding function might also be PI3K-dependent. (4) Membrane microdomains and caveolae are known to regulate Akt in a localized manner [55], and are associated with many aspects of lipid signaling including PI3K. Experimental testing and characterization of individual non-canonical factors is beyond the scope of this work, but will be the subject of future experiments.

Despite our assumptions, more than one significant non-canonical effect may be present. For example, we excluded the hypothesis of altered dephosphorylation (M4) because it wasn’t sufficient by itself to explain the data, but some type of altered dephosphorylation [48] could very well occur in conjunction with another mechanism. Another possibility we didn’t model is that a single non-canonical phenomenon could affect more than one step of the model. For example, pH could affect many steps of the pathway [56]. Finally, there could be other violations of our simplifying assumptions: for example, we interpreted the lack of PP2A in the membrane fraction to indicate that dephosphorylation is cytosolic in this cell type.

Another reason why we prohibited models with multiple non-canonical deviations is because each hypothetical effect (linear spline) increases the number of unknown parameters in the system, and worsens the risk of overfitting. When a model has too many unknown parameters, relative to the amount of data available for identifying the parameters, the model can become so under-determined that almost any hypothesis can fit the data, and nothing can be ruled out. On the other extreme, if we require the parameters to be identifiable from the data [57], then our model will become overly simplistic. Our modeling presents a case of under-determined models with 26 unknown parameters. Interestingly, the models were still useful, despite being under-determined (and implicitly, overfit), for a very important reason: **overfitting can only lead to errors with incorrect acceptance of an untrue model, not erroneous rejection of a true hypothesis.** Despite potential generosity to false hypotheses through overfitting, we were still able to reject most of the hypotheses after iteration with experiments.

The dynamic curve shape of Akt activity is important because mechanisms allowing normal cells to achieve a transient peak of extreme Akt activation could be mutated to provide a permanent state of extreme Akt activation in cancer. Akt is one of the most consistently over-activated pathways in cancer [58]. Another reason why the timing of Akt activation is important is because “hub” proteins (i.e., factors with many downstream targets and effectors) often achieve differential functions through the dynamics of activation, and not exclusively through the magnitude of activation [59,60]. This occurs for NF-kappaB [61], MAPK [62,63], p53 [64], and TGF-β/Smad signaling [65,66]. The effects of Akt on cell proliferation and cell cycle progression [67] have been found to depend on the timing, not just the magnitude, of Akt activation. We do not know the downstream functional implications of Akt overshoot dynamics in these cells, but it is logical to presume that membrane versus cytosolic localization of activated Akt could affect specificity. Akt has many targets at both membrane and cytosol, and altering the localization of activated Akt would alter the availability of active Akt toward different subsets of targets.

Textbook-level simplifications of signal transduction are necessarily incomplete, but to improve a model beyond the textbook level confronts an information overload, not only from the massive published literature, but also from the associated unknowns (e.g., reaction rates) that accompany each published discovery [68]. Exploratory experiments, chosen ad hoc, would consume enormous resources. The goal of our work was to use computational modelling within the most fundamental layer of biological decision-making: namely, constructing hypotheses and deciding what to test. Our chief result was to simulate alternative hypotheses (and their non-canonical deviations), and to use these simulations for investigating a temporal offset between upstream and downstream species (PIP3 and Aktp308) in Akt activation. Computational modeling clarified what information would be gained by each experiment, and extracted stronger inferences from the analysis of existing data. In the future, when hypothesis-evaluation becomes more automated, top-down coverage of alternative hypotheses can be integrated with customization of high-throughput cell-based assays, and with statistical methods for the optimal design of experiments [69], so that every step of the Scientific Method can occur in a high-dimensional space.

## Methods

### H0 model representation

To model the biochemical behavior of the system, we used a system of ODEs to describe the evolution of the species concentrations over time. Letting *s* denote the species and *e* denote which experiment or treatment condition is applied, the change of the transient concentration *x*_*s*,*e*_ over time can be represented in ODE form as

$$\frac{d}{dt}x_{s,e}\left(t,k\right)=f_{s,e}\left(x\left(t,k\right),i\left(t\right),k\right)$$ (1)

where *t* is the transient time point; **x**(*t*, **k**) is the set of species *x*_*s*,*e*_ (*t*, **k**) that evolve over time, governed by the equations of the system; **k** is the parameter vector including reaction rates and initial concentrations; *f*_*s*,*e*_ is an algebraic function derived from the biochemical reactions in the system, which may involve any parameters, species, inputs, or variable curves in the system; **i**(*t*) is the set of input species *i*_*s*,*e*_ (*t*) determined by predefined time-evolved functions rather than by the *f*_*s*,*e*_ equations. The species *x*_*s*,*e*_ (*t*, **k**) are experiment-dependent because the concentration trajectory of a species can be affected by altered experimental conditions, addition of inhibitors, etc. Although the inputs are not governed by the equations of the biochemical reactions, the inputs are also experiment-dependent to allow different conditions to cause different levels of inputs. For H0, **x** has six species [PDK1], [PIP3:PDK1m], [Aktc], [PIP3:Aktm], [Aktp308m], and [Aktp308c]. PIP3 is the only element of i. Because PIP3 is an input curve and not simulated by ODEs, we cannot simulate mass action binding, with depletion of unbound PIP3 as PIP3 forms a complex with PDK1m or Aktm. Instead, PIP3 appears as a simple multiplier in the equations describing the recruitment of PDK1 or Akt by PIP3 (Table 1, #1 and #3). The six ODEs for H0 are as follows:

$$\frac{d\left[\text{PDK}1\right]}{dt}=-k_{1}\left[\text{PIP}3\right]\left[\text{PDK}1\right]+k_{2}\left[\text{PIP}3:\text{PDK}1\text{m}\right]$$ (2)

$$\frac{d\left[\text{PIP}3:\text{PDK}1\text{m}\right]}{dt}=k_{1}\left[\text{PIP}3\right]\left[\text{PDK}1\right]-k_{2}\left[\text{PIP}3:\text{PDK}1\text{m}\right]$$ (3)

$$\frac{d\left[\text{Aktc}\right]}{dt}=-k_{3}\left[\text{PIP}3\right]\left[\text{Aktc}\right]+\frac{k_{7}\left[\text{Phosphatase}\right]\left[{\text{Aktp}}^{308}\text{c}\right]}{k_{8}+\left[{\text{Aktp}}^{308}\text{c}\right]}$$ (4)

$$\frac{d\left[\text{PIP}3:\text{Aktm}\right]}{dt}=k_{3}\left[\text{PIP}3\right]\left[\text{Aktc}\right]-\frac{k_{4}\left[\text{PIP}3:\text{PDK}1\text{m}\right]\left[\text{PIP}3:\text{Aktm}\right]}{k_{5}+\left[\text{PIP}3:\text{Aktm}\right]}$$ (5)

$$\frac{d\left[{\text{Aktp}}^{308}\text{m}\right]}{dt}=\frac{k_{4}\left[\text{PIP}3:\text{PDK}1\text{m}\right]\left[\text{PIP}3:\text{Aktm}\right]}{k_{5}+\left[\text{PIP}3:\text{Aktm}\right]}-k_{6}\left[{\text{Aktp}}^{308}\text{m}\right]$$ (6)

$$\frac{d\left[{\text{Aktp}}^{308}\text{c}\right]}{dt}=k_{6}\left[{\text{Aktp}}^{308}\text{m}\right]-\frac{k_{7}\left[\text{Phosphatase}\right]\left[{\text{Aktp}}^{308}\text{c}\right]}{k_{8}+\left[{\text{Aktp}}^{308}\text{c}\right]}$$ (7)

### Model simulation

Models were implemented and simulated using Matlab (v.7.6, The MathWorks, MA) with SBToolbox2 toolkit [70] and its SBPD extension package. *In silico* simulations used default settings for the ODE solver. Models were originally implemented in TextBC format (as required by SBToolbox2), which contains model states, model variables and model reactions information. SBML-formatted models are available in S1 Protocol.

### Parameter estimation for model H0

Whenever applicable, published data and kinetic rate constants [10,31] were adopted to ensure that the model would not violate biological knowledge. The remaining parameters were fitted to observed data in the process of model calibration. The parameter estimation process can be expressed as a sum-of-squared-error (SSE) optimization problem:

$$\underset{k}{\text{min}}\left\{\text{SSE}\right\}\equiv \underset{k}{\text{min}}\left\{\sum_{s}\sum_{e}\sum_{t}w_{s,e}\cdot {\left(x_{s,e}^{data}(t)-x_{s,e}(t,k)\right)}^{2}\right\}$$ (8)

where $x_{s,e}^{data}(t)$ is the experimental measure of species *s*, experiment *e*, time *t*, where $w_{s,e}=\frac{n_{t}}{\sum_{t}{\left(x_{s,e}^{data}(t)\right)}^{2}}$ is a time-independent inverted mean-square weight, calculated based on the observed data, and *n*_*t*_ is the number of time points [71]. Eq (8) describes the objective function for a variety of optimization methods [70,72,73] applied to any of our models, not just H0. Model fitting for the H0 model used 200 initial starts [74,75] for global search (Particle Swarm Optimization), followed by local search (Nelder-Mead). The algorithms were run using SBToolbox2 [70] with default settings.

### Using splines to represent non-canonical deviations

Each non-canonical deviation consists of a pseudo-species (a red oval in Fig 3), and a pseudo-reaction (a red dashed arrow in Fig 3).

Each pseudo-species is defined by a linear spline, which represents the strength of the unknown effect (literally, the abundance of the pseudo-species) at each point in time. A linear spline is a series of line segments connecting adjacent points called spline knots. We parameterized each spline using 7 spline knots representing the strength of the non-canonical effect at time t = 0, 2, 5, 10, 30, 60, and 120min. The spline knots are unknown variables, meaning that each pseudo-species starts out having unknown time-course.

A pseudo-reaction connects each pseudo-species to the rest of the pathway. Like any biochemical reaction, a pseudo-reaction is described using the ordinary differential equations (ODEs) of the model. The pseudo-species appear in the ODEs as input species, similar to PIP3. In mathematical terms, an input species appears in the right-hand side of the ODEs but not the left hand side. In practical terms, an input species can drive biochemical reactions in the ODEs by serving as a reagent, but it is not governed by the ODEs, meaning the evolution of its level over time is not defined by solving the ODEs.

The spline knots are unknown variables, so they are treated like any other unknown parameter during the model fitting process. Therefore the spline curve gets adjusted and optimized by the parameter estimation phase, so as to maximize the fit of the model to the data. In other words, the parameter optimization process serves to estimate how much of the non-canonical effect would occur at each timepoint, in order to allow the overall model (including the non-canonical effect) to agree with the data.

### The alternative models M1 –M5

Each alternative model was constructed by adding a non-canonical deviation (S1 Text) to the canonical pathway model H0 (Eqs 1–7). Each non-canonical deviation (a red addition in Fig 3) consists of a pseudo-species and a pseudo-reaction. The pseudo-species are defined by splines, as described above, and the pseudo-reactions are described below as alterations to *f*, the set of ODEs in the original H0 model. Although the splines **v** of the pseudo-species appear in the ODEs as “input species” and are not governed by the differential equations, they are still functions of the parameter vector **k** because they are defined by the spline knots, which are variables in the parameter vector **k**. Therefore, the alternative models with non-canonical deviations can be represented with the following equation instead of Eq (1):

$$\frac{d}{dt}x_{s,e}\left(t,k\right)=f_{s,e}\left(x\left(t,k\right),i\left(t\right),v\left(t,k\right),k\right)$$ (9)

where **v**(*t*, **k**) is the linear spline for the pseudo-species of the non-canonical deviation, and the differential equations *f* are modified as follows. The equations for Model M1 are the same as for Model H0 except with Eqs (10 and 11) in place of Eqs (4 and 5).

$$\frac{d\left[\text{Aktc}\right]}{dt}=-k_{3}\left[\text{PIP}3\right]\left[\text{Recruitment}\right]\left[\text{Aktc}\right]+\frac{k_{7}\left[\text{Phosphatase}\right]\left[{\text{Aktp}}^{308}\text{c}\right]}{k_{8}+\left[{\text{Aktp}}^{308}\text{c}\right]}$$ (10)

$$\frac{d\left[\text{PIP}3:\text{Aktm}\right]}{dt}=k_{3}\left[\text{PIP}3\right]\left[\text{Recruitment}\right]\left[\text{Aktc}\right]-\frac{k_{4}\left[\text{PIP}3:\text{PDK}1\text{m}\right]\left[\text{PIP}3:\text{Aktm}\right]}{k_{5}+\left[\text{PIP}3:\text{Aktm}\right]}$$ (11)

M2 is also based on model H0 but with Eqs (12 and 13) instead of Eqs (4 and 5).

$$\frac{d\left[\text{Aktc}\right]}{dt}=-k_{3}\left[\text{PIP}3\right]\left[\text{Aktc}\right]+\frac{k_{7}\left[\text{Phosphatase}\right]\left[{\text{Aktp}}^{308}\text{c}\right]}{k_{8}+\left[{\text{Aktp}}^{308}\text{c}\right]}-k_{9}\left[\text{Recruitment}\right]\left[\text{Aktc}\right]$$ (12)

$$\frac{d\left[\text{PIP}3:\text{Aktm}\right]}{dt}=k_{3}\left[\text{PIP}3\right]\left[\text{Aktc}\right]-\frac{k_{4}\left[\text{PIP}3:\text{PDK}1\text{m}\right]\left[\text{PIP}3:\text{Aktm}\right]}{k_{5}+\left[\text{PIP}3:\text{Aktm}\right]}+k_{9}\left[\text{Recruitment}\right]\left[\text{Aktc}\right]$$ (13)

Model M3 has a change in species as well as equations. M3 is the same as H0 except for Eqs (6 and 7) being replaced by Eqs (14–16), and species Aktp^308^m being replaced by a pair of species Aktp^308^mf (representing membrane Aktp^308^ that is free to dissociate from the membrane) and Aktp^308^mt (membrane Aktp^308^ that is trapped at the membrane).

$$\begin{array}{l} \frac{d\left[{\text{Aktp}}^{308}\text{mf}\right]}{dt}=\frac{k_{4}\left[\text{PIP}3:\text{PDK}1\text{m}\right]\left[\text{PIP}3:\text{Aktm}\right]}{k_{5}+\left[\text{PIP}3:\text{Aktm}\right]}-k_{6}\left[{\text{Aktp}}^{308}\text{mf}\right]\\ \;-k_{10}\left[\text{Retention}\right]\left[{\text{Aktp}}^{308}\text{mf}\right]+k_{11}\left[{\text{Aktp}}^{308}\text{mt}\right] \end{array}$$ (14)

$$\frac{d\left[{\text{Aktp}}^{308}\text{mt}\right]}{dt}=k_{10}\left[\text{Retention}\right]\left[{\text{Aktp}}^{308}\text{m}\;\text{f}\right]-k_{11}\left[{\text{Aktp}}^{308}\text{m}\;\text{t}\right]$$ (15)

$$\frac{d\left[{\text{Aktp}}^{308}\text{c}\right]}{dt}=k_{6}\left[{\text{Aktp}}^{308}\text{m}\;\text{f}\right]-\frac{k_{7}\left[\text{Phosphatase}\right]\left[{\text{Aktp}}^{308}\text{c}\right]}{k_{8}+\left[{\text{Aktp}}^{308}\text{c}\right]}$$ (16)

Model M4 is based on model H0 except with additional Eqs (17 and 18).

$$\frac{d\left[\text{Phosphatase}\right]}{dt}=-k_{12}\left[\text{Phosphatase}\right]+k_{13}\left[\text{InaccPhosphatase}\right]$$ (17)

$$\frac{d\left[\text{InaccPhosphatase}\right]}{dt}=k_{12}\left[\text{Phosphatase}\right]-k_{13}\left[\text{InaccPhosphatase}\right]$$ (18)

Model M5 is based on model H0 but with Eqs (19 and 20) instead of Eqs (5 and 6).

$$\frac{d\left[\text{PIP}3:\text{Aktm}\right]}{dt}=k_{3}\left[\text{PIP}3\right]\left[\text{Aktc}\right]-\frac{k_{4}\left[\text{PIP}3:\text{PDK}1\text{m}\right]\left[\text{Phosphorylation}\right]\left[\text{PIP}3:\text{Aktm}\right]}{k_{5}+\left[\text{PIP}3:\text{Aktm}\right]}$$ (19)

$$\frac{d\left[{\text{Aktp}}^{308}\text{m}\right]}{dt}=\frac{k_{4}\left[\text{PIP}3:\text{PDK}1\text{m}\right]\left[\text{Phosphorylation}\right]\left[\text{PIP}3:\text{Aktm}\right]}{k_{5}+\left[\text{PIP}3:\text{Aktm}\right]}-k_{6}\left[{\text{Aktp}}^{308}\text{m}\right]$$ (20)

In additional to the rate parameters *k*_1_-*k*_8_ from the canonical model (Table 1), the alternative models (M1-M5) include new parameters *k*_9_-*k*_13_, the values of which must be estimated during the parameter estimation process. The Phosphatase level was held constant for all models except M5.

### Parameter estimation for the alternative models

Each model is fit to the data using parameter estimation. As shown in the flowchart of Fig 3F, parameter estimation occurs by performing many iterations of simulating the model (Box 1) while changing the values of the variable parameters. In that way, parameter estimation for the alternative models is exactly similar to parameter estimation for H0, although the parameter vectors were longer because they contained additional parameters for the reaction rates of the pseudo-reactions and the spline knots of the pseudo-species.

The convergence of parameter estimation depends in general on the numerical stability of the function being optimized. Therefore, in our use of splines to represent non-canonical deviations (see above), we chose to use linear splines, despite their jagged appearance, because they have better numerical stability than smooth splines.

We used the following multiple-fit strategy for parameter estimation of the alternative models. After randomly initializing the unknown variable parameters, global optimization was performed using Particle Swarm Optimization (PSO) [74], followed by local refinement with the Nelder-Mead (NM) algorithm [75]. The PSO+NM optimization process was repeated 100 times, applying exponential noise (alpha = 0.5) to the initial guess of all parameters at each iteration. During PSO+NM optimization, the quality of fit was judged by sum-of-squared error. Finally we plotted and examined the models manually. If previous steps failed to produce a good fit with the data, we tried additional manual tuning of the parameters based on human intuition. Manual tuning means adjusting some parameters values by hand. Each instance of manual tuning was always followed by Nelder-Mead local optimization, and optionally by additional iterations of stochastic search methods such as PSO+NM optimization. In practice, we found manual tuning to be an important contribution to parameter estimation, because when automated search methods got stuck in local minima, they sometimes had stark violations of the data, but stochastic restarts could require a huge number of random trials before finding the right direction for a leap. In contrast, a human could “understand” which direction to bump the parameter vector, so the automated optimization could quickly descend into a better local min. The search process terminated when parameter estimation reached a local min, and when stochastic restarts failed to improve on the min, and when manual tuning followed by Nelder-Mead failed to improve on the min. After the optimization phase, the quality of fit was scored using peak time, peak width, and peak magnitude.

### Cell culture experiments

#### Cell culture

MEF^WT^ cells were maintained in Dulbecco’s modified Eagle’s medium/high glucose (DMEM) supplemented with 2mM L-glutamine and 1mM gentamicin. Cells were incubated in a 5% CO_2_ incubator at 37°C. Cells were grown in DMEM containing 10% fetal bovine serum (FBS). Serum starved cells were grown for 24hrs in DMEM containing 0.5% FBS before being exposed to 10% FBS for the indicated time.

#### Western blot

Cells were lysed in the lysis buffer (50mM Tris-HCl pH7.4, 150mM NaCl, 1mM EGTA, 1mM EDTA, 0.5% (v/v) Triton X-100) containing 1mM PMSF, 5μg/ml leupeptin, 5μg/ml pepstatin A, 1μg/ml aprotinin and 1mM sodium orthovanadate. Cell lysates were then subjected to SDS-PAGE.

#### PIP3 measurement

Immunodetection of PIP3 level and confocal laser scanning microscopy was performed as described in [26].

#### Data normalization

Raw data were normalized before model fitting, because experimental data were acquired by different assays (western blotting, confocal imaging). For each measurable species, time series data were rescaled using the same normalization scheme (defined by the scale and offset values) for all experiments. For membrane fractionation experiments, data were normalized such that the concentration of a membrane fraction was never greater than that of total cell lysate.

## Supporting Information

S1 Fig Western blots for PDK1 in the membrane fraction in time-series. The cadherin controls are repeated for ease of comparison.
(PDF)

S2 Fig Top-down coverage of the pathway for investigating an unknown effect. Five events (dashed boxes) in the canonical PIP3/Akt cascade were identified: PDK1 recruitment, Akt recruitment, Akt Thr^308^ phosphorylation, Akt membrane-cytosol translocation and Akt Thr^308^ dephosphorylation. One event (PDK1 recruitment) was rejected based on existing measurements showing that PDK1m mimics PIP3 dynamics.
(PDF)

S3 Fig (A) Time-series measurements of total Akt in the cytosol showed (B) qualitative disagreement with simulations of the retention model, suggesting that the retention model alone is insufficient to explain the data. (PDF)

S1 Dataset Dataset and normalization procedures for the membrane fractionation experiments. **(A)** Immunoblots of the membrane fraction of Aktp^308^ (Aktp^308^m) and total Akt (TotMemAkt) in serum-stimulated mouse embryonic fibroblasts (MEFs). In the first three sets of replica (Repeat 1–3), measurements were taken for Aktp^308^m, TotMemAkt and Cadherin. In the latter three sets of replica (Repeat 4–6), measurements were taken for TotMemAkt and Cadherin. Cells were grown initially in 10% fetal bovine serum (FBS), then incubated 24 hours in culture medium containing 0.5% FBS. Then 10% FBS was re-introduced (“serum stimulation”) and subsequent measurements were taken, relative to the serum stimulation. Time t = 0 indicates cells in 0.5% FBS without serum stimulation. **(B)** The procedure for producing the summary plot in Fig 5B is described verbally, and numerically by showing each intermediate step of the calculation. Raw densitometry was calculated based on each band intensity from western blot images. The Aktp^308^m and TotMemAkt values were normalized by the Cadherin values, and converted to fold-change from t = 0min. Increments were calculated from the normalized fold-change values, and median increments were derived. Finally, the “median values” calculated from the median increments were rescaled so that the largest values for the Aktp^308^m and TotMemAkt time-series are 5 and 10, respectively.
(PDF)

S1 Tables Quantified scores of the alternative models (M1-M5) against the experimental data, according to the peak features. The red row corresponds to the Σ(err) numbers shown in Fig 6K–6O. The bold black row “Sum (overall)” is the total score of each model against the data.
(PDF)

S1 Document Ensemble analysis, scoring families of models for M1, M2 and M3. (PDF)

S1 Protocol Zipfile containing all models in SBML format and SBToolbox format. (ZIP)

S1 Text Construction of the alternative models M1-M5. (PDF)
