# Supplementary material for: Non-canonical Activation of Akt in Serum-Stimulated Fibroblasts, Revealed by Comparative Modeling of Pathway Dynamics
Source: PLoS Comput Biol. 2015 Nov 10;11(11):e1004505. doi: 10.1371/journal.pcbi.1004505 (PMC4640559; doi:10.1371/journal.pcbi.1004505)

Supplemental Material for "Non-Canonical Activation of Akt in Serum-stimulated Fibroblasts, Revealed by Comparative Modeling of Pathway Dynamics"

Supplemental Figure S1. Western blots for PDK1 in the membrane fraction in time-series. The cadherin controls are repeated for ease of comparison.

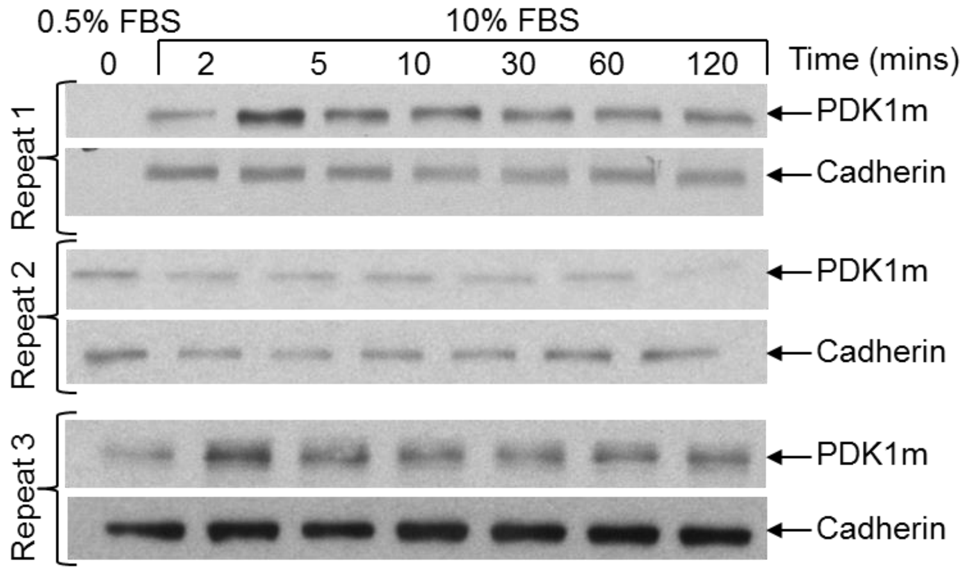

Supplement: S1 Fig — The cadherin controls are repeated for ease of comparison. (PDF) [file pcbi.1004505.s001.pdf]
