# Supplementary material for: Non-canonical Activation of Akt in Serum-Stimulated Fibroblasts, Revealed by Comparative Modeling of Pathway Dynamics
Source: PLoS Comput Biol. 2015 Nov 10;11(11):e1004505. doi: 10.1371/journal.pcbi.1004505 (PMC4640559; doi:10.1371/journal.pcbi.1004505)
Supplement: S2 Fig — Five events (dashed boxes) in the canonical PIP3/Akt cascade were identified: PDK1 recruitment, Akt recruitment, Akt Thr308 phosphorylation, Akt membrane-cytosol translocation and Akt Thr308 dephosphorylation. One event (PDK1 recruitment) was rejected based on existing measurements showing that PDK1m mimics PIP3 dynamics. (PDF) [file pcbi.1004505.s002.pdf]

Supplemental Material for “Non-Canonical Activation of Akt in Serum-stimulated Fibroblasts, Revealed by Comparative Modeling of Pathway Dynamics”

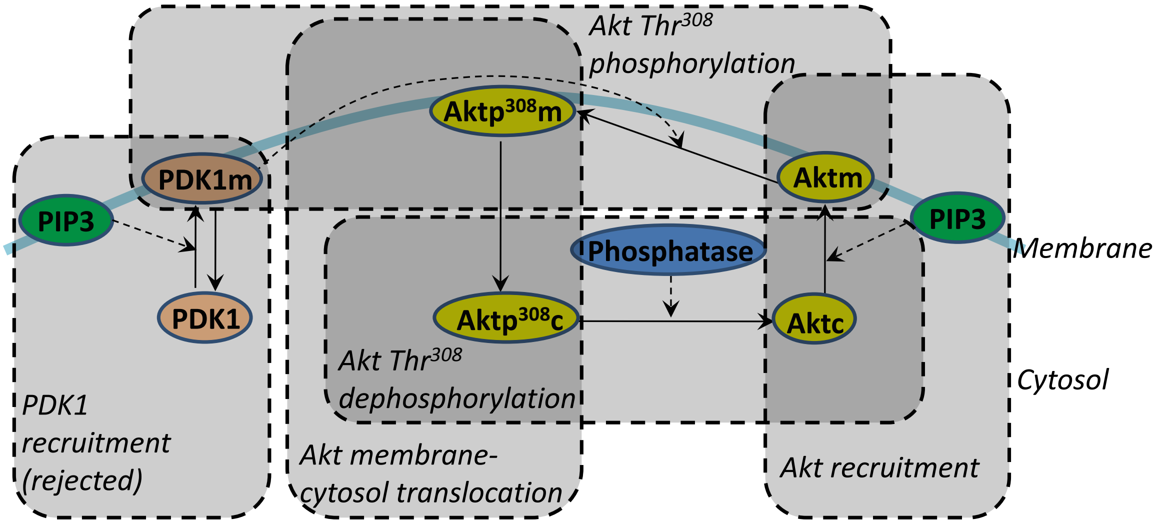

Supplemental Figure S2. Top-down coverage of the pathway for investigating an unknown effect. Five events (dashed boxes) in the canonical PIP3/Akt cascade were identified: PDK1 recruitment, Akt recruitment, Akt Thr^{308} phosphorylation, Akt membrane-cytosol translocation and Akt Thr^{308} dephosphorylation. One event (PDK1 recruitment) was rejected based on existing measurements showing that PDK1m mimics PIP3 dynamics.
