# Supplementary material for: Non-canonical Activation of Akt in Serum-Stimulated Fibroblasts, Revealed by Comparative Modeling of Pathway Dynamics
Source: PLoS Comput Biol. 2015 Nov 10;11(11):e1004505. doi: 10.1371/journal.pcbi.1004505 (PMC4640559; doi:10.1371/journal.pcbi.1004505)
Supplement: S3 Fig — (PDF) [file pcbi.1004505.s003.pdf]

Supplemental Material for “Non-Canonical Activation of Akt in Serum-stimulated Fibroblasts, Revealed by Comparative Modeling of Pathway Dynamics”

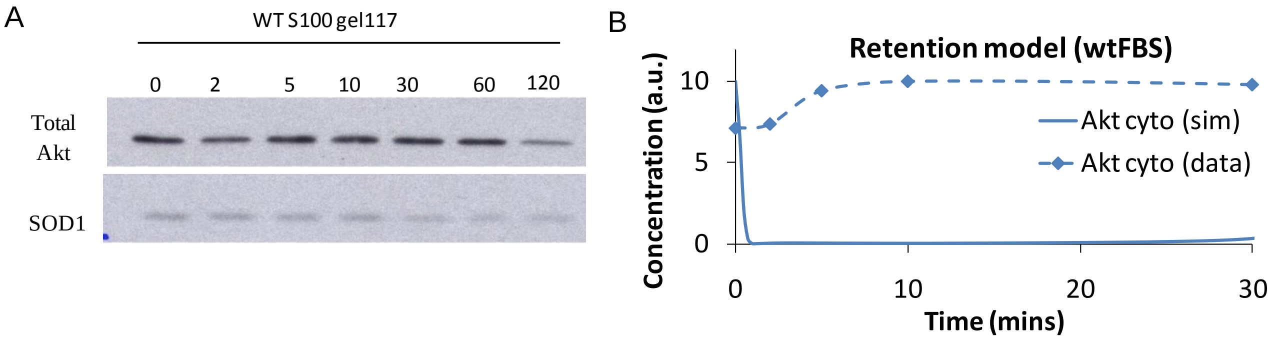

Supplemental Figure S3. (A) Time-series measurements of total Akt in the cytosol showed (B) qualitative disagreement with simulations of the retention model, suggesting that the retention model alone is insufficient to explain the data.
