# Supplementary material for: Non-canonical Activation of Akt in Serum-Stimulated Fibroblasts, Revealed by Comparative Modeling of Pathway Dynamics
Source: PLoS Comput Biol. 2015 Nov 10;11(11):e1004505. doi: 10.1371/journal.pcbi.1004505 (PMC4640559; doi:10.1371/journal.pcbi.1004505)
Supplement: S1 Dataset — (A) Immunoblots of the membrane fraction of Aktp308 (Aktp308m) and total Akt (TotMemAkt) in serum-stimulated mouse embryonic fibroblasts (MEFs). In the first three sets of replica (Repeat 1–3), measurements were taken for Aktp308m, TotMemAkt and Cadherin. In the latter three sets of replica (Repeat 4–6), measurements were taken for TotMemAkt and Cadherin. Cells were grown initially in 10% fetal bovine serum (FBS), then incubated 24 hours in culture medium containing 0.5% FBS. Then 10% FBS was re-introduced (“serum stimulation”) and subsequent measurements were taken, relative to the serum stimulation. Time t = 0 indicates cells in 0.5% FBS without serum stimulation. (B) The procedure for producing the summary plot in Fig 5B is described verbally, and numerically by showing each intermediate step of the calculation. Raw densitometry was calculated based on each band intensity from western blot images. The Aktp308m and TotMemAkt values were normalized by the Cadherin values, and converted to fold-change from t = 0min. Increments were calculated from the normalized fold-change values, and median increments were derived. Finally, the “median values” calculated from the median increments were rescaled so that the largest values for the Aktp308m and TotMemAkt time-series are 5 and 10, respectively. (PDF) [file pcbi.1004505.s004.pdf]

Supplemental Material for “Non-Canonical Activation of Akt in Serum-stimulated Fibroblasts, Revealed by Comparative Modeling of Pathway Dynamics”

Supplemental Dataset S1. Dataset and normalization procedures for the membrane fractionation experiments. (A) Immunoblots of the membrane fraction of $Aktp^{308}$ ($Aktp^{308}m$) and total Akt (TotMemAkt) in serum-stimulated mouse embryonic fibroblasts (MEFs). In the first three sets of replica (Repeat 1-3), measurements were taken for $Aktp^{308}m$, TotMemAkt and Cadherin. In the latter three sets of replica (Repeat 4-6), measurements were taken for TotMemAkt and Cadherin. Cells were grown initially in 10% fetal bovine serum (FBS), then incubated 24 hours in culture medium containing 0.5% FBS. Then 10% FBS was re-introduced (“serum stimulation”) and subsequent measurements were taken, relative to the serum stimulation. Time t=0 indicates cells in 0.5% FBS without serum stimulation. **(B)** Tables with quantified time-series abundance. Raw intensity was obtained from densitometry of each band from Western blot images. The $Aktp^{308}m$ and TotMemAkt raw intensity values were normalized by the Cadherin values and converted to fold-change from t=0min. Increments were calculated from the normalized fold-change values, and median increments were derived for each timepoint. Finally, the “median values” calculated from the median increments were rescaled so that the largest values for the $Aktp^{308}m$ and TotMemAkt time-series are 5 and 10, respectively. **(C)** Fractionation controls for the 3 $Aktp^{308}m$ experiments shown in (A). The same biological samples have been run on another electrophoresis gel with the cytosolic and membrane fractions shown side-by-side. SOD1 (superoxide dismutase 1) is a marker for the cytosolic fraction and Cadherin is a marker for the membrane fraction.

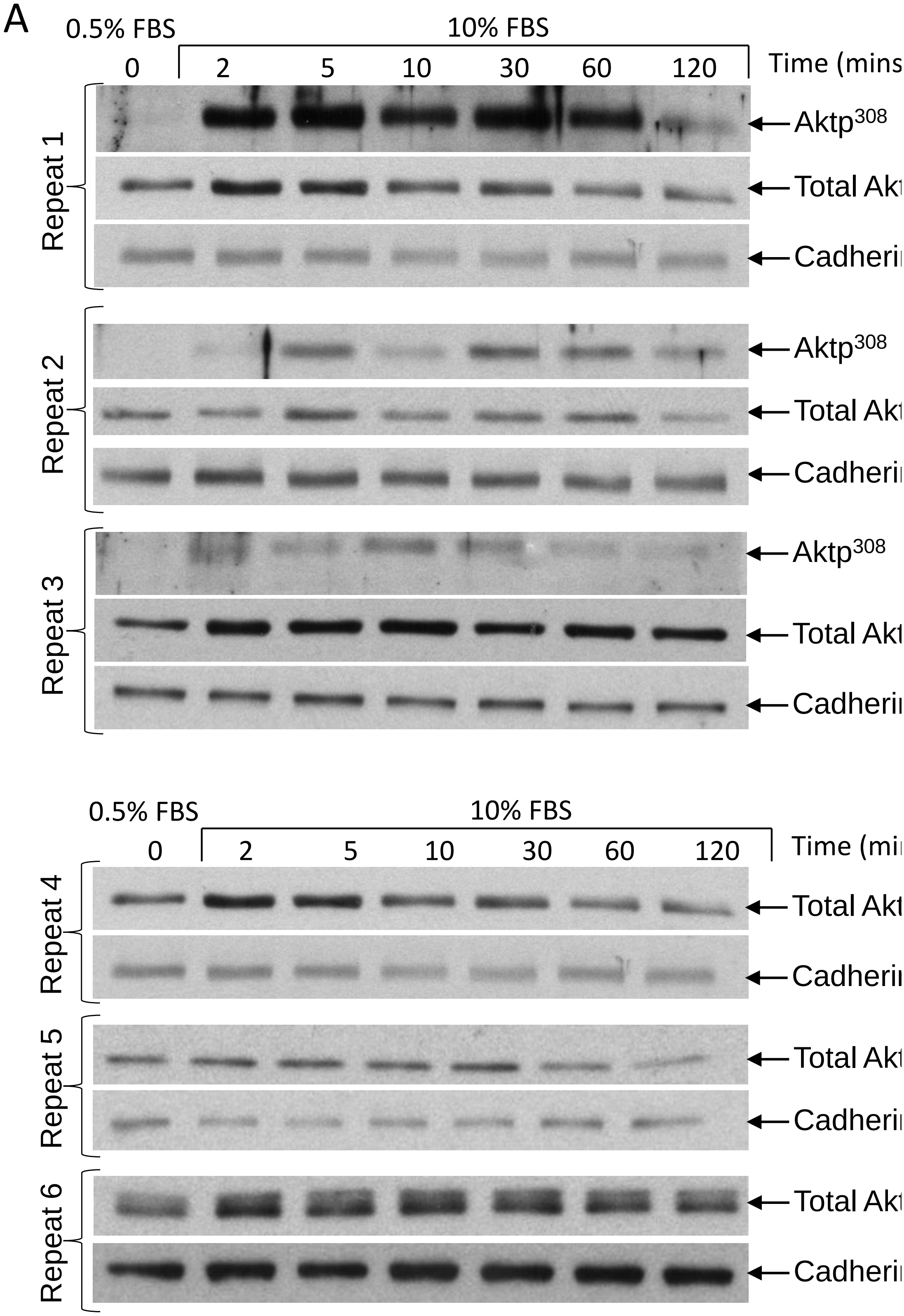
A
0.5% FBS
10% FBS
0
2
5
10
30
60
120
Time (mins
Repeat 1
Repeat 2
Repeat 3
Aktp308
Total Ak
Cadheri
Time (mi
Repeat 4
Repeat 5
Repeat 6

C

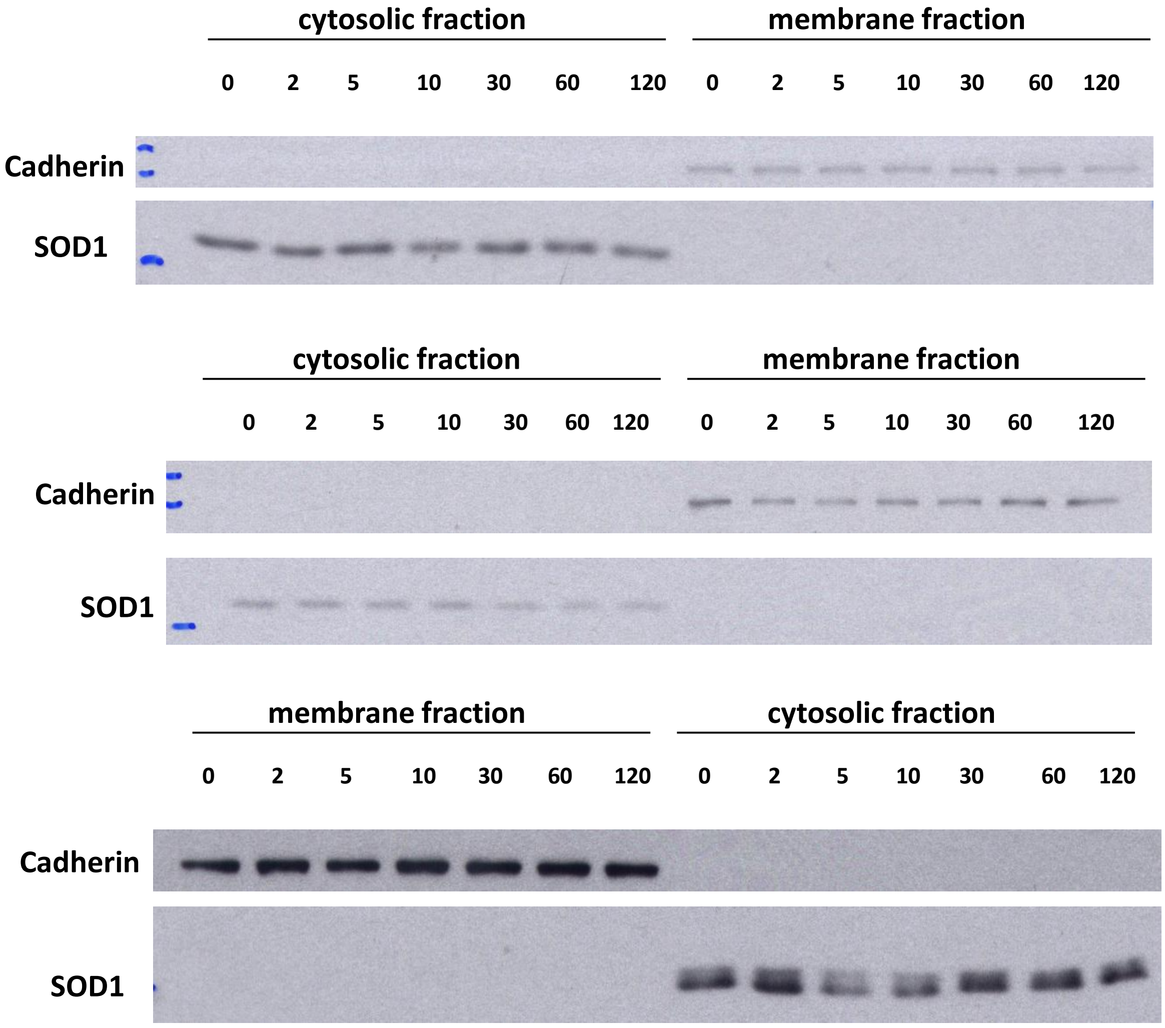
cytosolic fraction
membrane fraction
0 2 5 10 30 60 120 0 2 5 10 30 60 120
Cadherin
SOD1
cytosolic fraction
membrane fraction
0 2 5 10 30 60 120 0 2 5 10 30 60 120
Cadherin
SOD1
membrane fraction
cytosolic fraction
0 2 5 10 30 60 120 0 2 5 10 30 60 120
Cadherin
SOD1
