# Supplementary material for: Non-canonical Activation of Akt in Serum-Stimulated Fibroblasts, Revealed by Comparative Modeling of Pathway Dynamics
Source: PLoS Comput Biol. 2015 Nov 10;11(11):e1004505. doi: 10.1371/journal.pcbi.1004505 (PMC4640559; doi:10.1371/journal.pcbi.1004505)
Supplement: S1 Tables — The red row corresponds to the Σ(err) numbers shown in Fig 6K–6O. The bold black row “Sum (overall)” is the total score of each model against the data. (PDF) [file pcbi.1004505.s005.pdf]

Supplemental Material for "Non-Canonical Activation of Akt in Serum-stimulated Fibroblasts, Revealed by Comparative Modeling of Pathway Dynamics"

Supplemental Tables S1. Quantified scores of the alternative models (M1-M5) against the experimental data, according to the peak features. The red row corresponds to the Σ(err) numbers shown in Fig 6K-O. The bold black row "Sum (overall)" is the total score of each model against the data.

		Ratio with data				
		**M1**	**M2**	**M3**	**M4**	**M5**
Peak time	PDK1m	1.2	1.08	1.08	1.08	1.2
	$Aktp^{308}$	1.248	1.176	0.864	0.928	1.032
	Akt mem	0.912	0.864	0.24	0.192	0.432
	$Aktp^{308}$ mem	0.864	0.232	0.824	0.128	0.448
Peak amplitude	PDK1m	0.8583125	1.01935	0.5918375	0.6076125	0.8583875
	$Aktp^{308}$	0.61169	0.63799	0.90322	0.93289	0.85558
	Akt mem	1.0153	1.0372	1.42635714	1.41864286	1.42167143
	$Aktp^{308}$ mem	0.73206	0.74078	1.77748	0.38164	0.9218
Peak width	PDK1m	0.92307692	0.92307692	0.64615385	0.64615385	0.92307692
	$Aktp^{308}$	2.50810811	3.00540541	1.98918919	1.85945946	2.81081081
	Akt mem	0.11662404	0.11662404	1.38721228	0.07365729	0.22710997
	$Aktp^{308}$ mem	0.32181818	0.40363636	0.43636364	0.06	0.21818182
SSE (Peak time)		0.127744	0.645696	0.633472	1.424832	0.668352
SSE (Peak amplitude)		0.24288594	0.20000451	0.96221856	0.71610263	0.22483327
SSE (Peak width)		3.52059089	5.16357067	1.57132165	2.60558848	4.49355161
SSE Score (Overall)		**3.89122083**	**6.00927118**	**3.16701221**	**4.74652311**	**5.38673688**

SSE = sum-of-square error =SUM((ratio-1)^2))

		Absolute difference with data				
		**M1**	**M2**	**M3**	**M4**	**M5**
Peak time	PDK1m	0.4	0.16	0.16	0.16	0.4
	$Aktp^{308}$	7.44	5.28	4.08	2.16	0.96
	Akt mem	0.44	0.68	3.8	4.04	2.84
	$Aktp^{308}$ mem	4.08	23.04	5.28	26.16	16.56
Peak amplitude	PDK1m	1.1335	0.1548	3.2653	3.1391	1.1329
	$Aktp^{308}$	3.8831	3.6201	0.9678	0.6711	1.4442
	Akt mem	0.1071	0.2604	2.9845	2.9305	2.9517
	$Aktp^{308}$ mem	1.3397	1.2961	3.8874	3.0918	0.391
Peak width	PDK1m	0.2	0.2	0.92	0.92	0.2
	$Aktp^{308}$	16.74	22.26	10.98	9.54	20.1
	Akt mem	34.54	34.54	15.14	36.22	30.22
	$Aktp^{308}$ mem	29.84	26.24	24.8	41.36	34.4
Sum (Peak time)		12.36	29.16	13.32	32.52	20.76
Sum (Peak amplitude)		6.4634	5.3314	11.105	9.8325	5.9198
Sum (Peak width)		81.32	83.24	51.84	88.04	84.92
Sum (Overall)		**100.1434**	**117.7314**	**76.265**	**130.3925**	**111.5998**

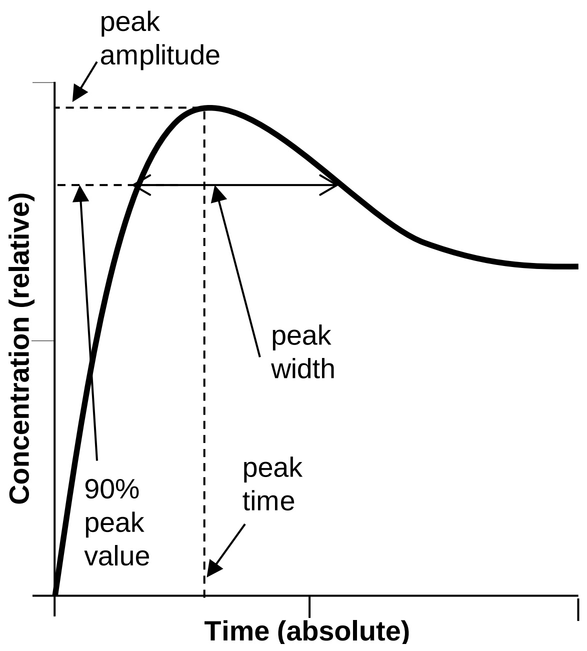

Peak Vector

The "peak vector" is a quantification of the dynamics of each system based on the properties of the major peak in the time course: peak time (in minutes), peak amplitude (defined as the fold-change difference of the peak level versus the steady-state level), and peak width. Peak width is defined as the interpolated width of the curve at 90% of its maximum. The 90% level was chosen so that every curve would contain a peak. Note that the peak features are uniquely defined for each time-series experiment and each theoretical simulation. The peak features provide a simple abstraction for the dynamics of each trajectory, based on the properties of the highest peak.

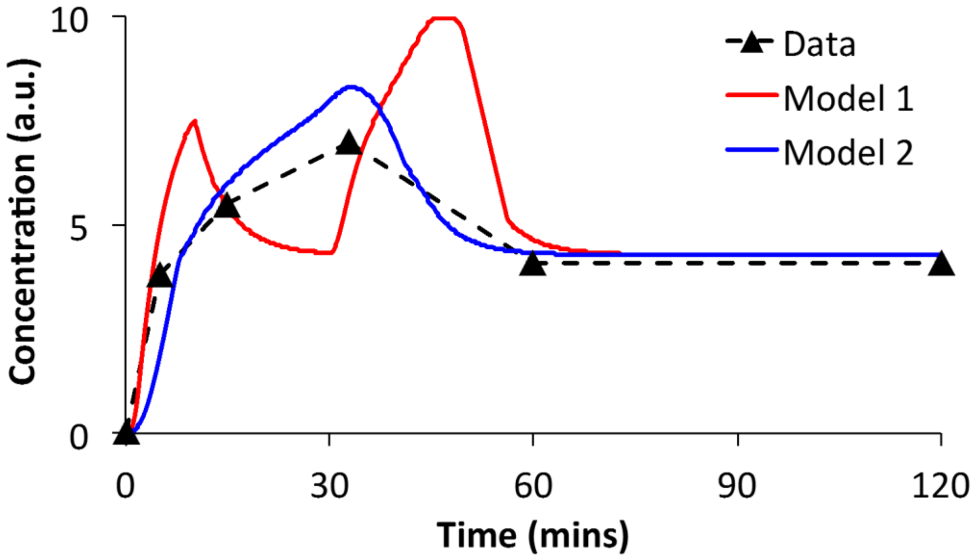

Problems and pitfalls with scoring methods

The black triangles above represent a time-series dataset. Model 1 and Model 2 are two different computational simulations of the Akt pathway which show different types of agreement/disagreement with the data, and it's not necessarily obvious what a "good" scoring method should do. Because the approximate trend of the black triangles has been observed by many labs in different contexts, some people might believe the experimental data were more likely generated by a system with a gradual main peak, not by a system with multiple-peak oscillation, but the prior expectations are subject to debate. If there is a prior expectation for a simpler trajectory with fewer peaks, then the multi-peak trajectory of model 1 (red curve) would be viewed as over-fitted. When scoring peak features, the blue curve is judged to be a better fit than the red curve. If we instead compute the sum of squared absolute error (SSE) for each timepoint, then model 1 (red) can give almost perfect agreement with each experimental timepoint, much better than the blue curve. Any scoring method has strengths and weaknesses. Scoring the characteristics of the major peak can give pathological results if there are two nearly-equal peaks.
