# Supplementary material for: Non-canonical Activation of Akt in Serum-Stimulated Fibroblasts, Revealed by Comparative Modeling of Pathway Dynamics
Source: PLoS Comput Biol. 2015 Nov 10;11(11):e1004505. doi: 10.1371/journal.pcbi.1004505 (PMC4640559; doi:10.1371/journal.pcbi.1004505)
Supplement: S1 Document — (PDF) [file pcbi.1004505.s006.pdf]

Supplemental Material for "Non-Canonical Activation of Akt in Serum-stimulated Fibroblasts, Revealed by Comparative Modeling of Pathway Dynamics"

Supplemental Document S1. Ensemble analysis, scoring families of models for M1, M2 and M3.

With the complexity and potential lack of well-defined minima in the alternative models, it was possible that multiple models within each alternative hypothesis could match the data equally well. Modeling studies such as by Chen *et al*. have considered multiple fits for evaluating model behavior rather than relying on a single best model (Chen, et al., 2009).

Following this approach, we proceeded to evaluate a broad spectrum of individuals. We generated an ensemble of parameterizations for each set of ODEs, which in practice means a family of similar instances for each alternative model. In each family, each of the 26 nominal parameters (*i.e*. best estimates from the optimal recruitment or retention model obtained by multiple-fitting described in the previous sections) was one-at-a-time doubled or halved, generating additional 52 slightly modified individuals surrounding the optimal model (i.e. model with nominal parameters).

In the figure below, the normalized peak features computed from these simulations (solid colored lines) were plotted in a radar chart against the peak features of the experimental dataset (thick dashed black lines), with the experimentally observed levels defined to be along the unit circle. See the table below of peak feature scores ("ratio with data") for many instances of each model. We propose this type of multi-factor scoring and plot to display the trade-off in fitness between different parameterizations of the same ODEs, and we hope future work in pathway modeling will consider reuse of this visualization. The variation among members of each model family suggests the ruggedness of the peak score in the parameter space around the nominal parameters.

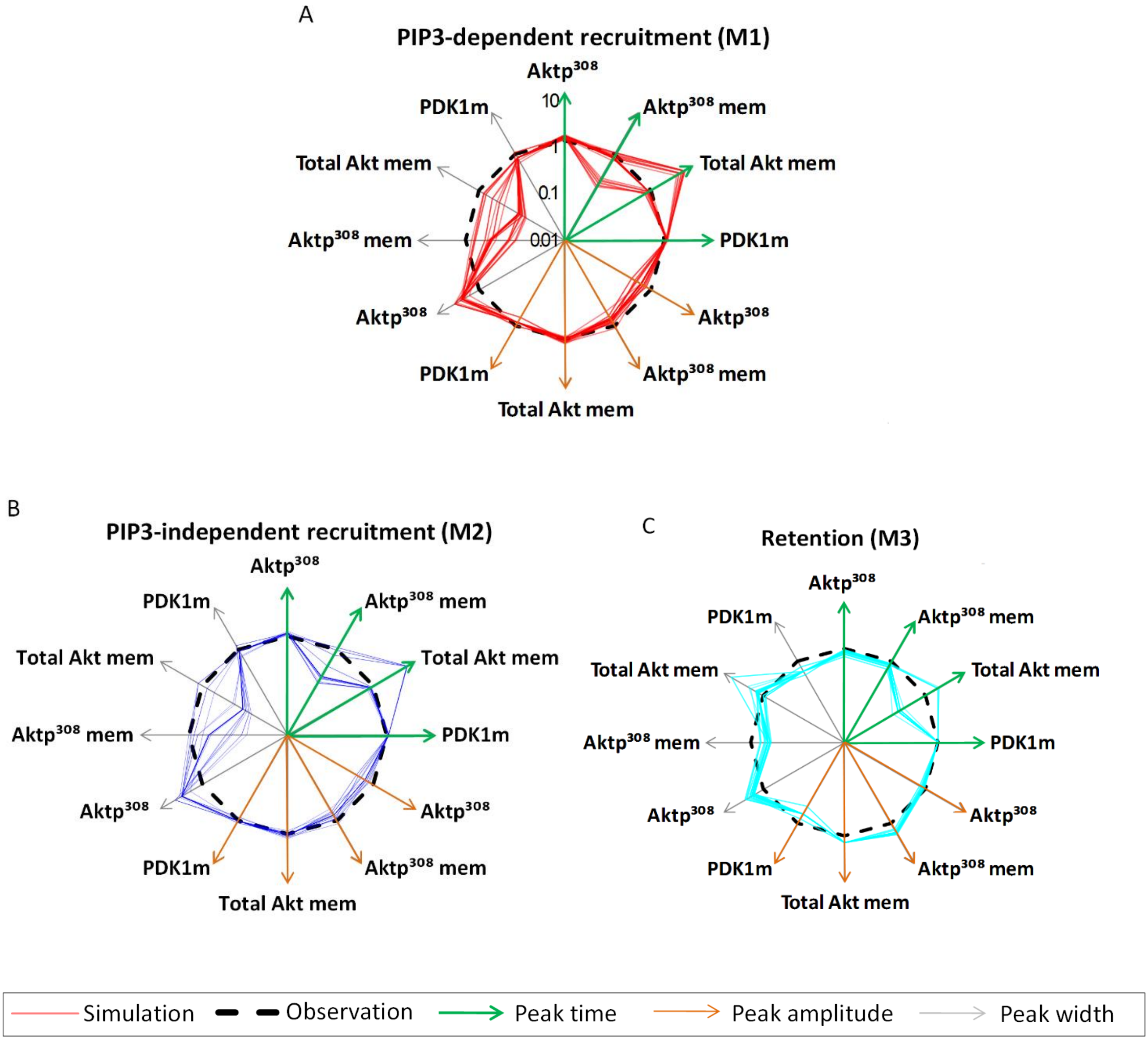

Figure for Text S6. (A) A diagram illustrating the definitions of the peak properties. Peak width is defined as the difference between the times when the level reaches 90% of the peak value in the time course. (B-D) A novel visualization scheme for displaying a multi-factorial comparison between an experimental dataset and a family of models. In these comparisons, 3 peak properties are used for comparing each of 4 measured species (3*4=12 axes). In each radar chart plot, four green axis indicate the peak times of the four measured species. Four brown axes indicate the peak amplitude and four gray axes indicate the peak width of the four measured species. The thickly dashed black circle indicates the peak vector of measurements, which is normalized to a unit vector. Multiple individuals of (B) PIP3-dependent recruitment, (C) retention, and (D) PIP3-independent model families (53 models per family) were compared with respect to the 12-dimensional peak vectors. Each model family contains the nominal parameters with one parameter doubled or halved. Peak time, peak amplitude and peak width were groups into arcs of the circle marked by colored arrows. Each arc contains four axes corresponding to the four measured time-series. All peak vectors from simulation (solid thin lines) were normalized with respect to the peak vector from measured data (dashed black circle) before plotting.

		Ratio with data (best=1)									
		M1-1	M1-2	M1-3	M1-4	M1-5	M1-6	M1-7	M1-8	M1-9	M1-10
Peak time	$Aktp^{308}$	1.25333333	1.29333333	1.21	1.31333333	1.26333333	1.15666667	1.17333333	1.27666667	1.25666667	1.25333333
	$Aktp^{308}$ mem	0.86666667	0.89666667	0.84666667	1.05333333	0.96666667	0.18333333	1.05	0.25	0.86333333	0.86666667
	Total Akt mem	0.9	5.14	0.88	6.04	5.92	0.82	6.04	0.88	0.9	0.9
	PDK1m	1.15	1.25	1.25	1.15	1.15	1.15	1.15	1.15	1.15	1.15
Peak amplitude	$Aktp^{308}$	0.61166	0.47867	0.6807	0.47968	0.36361	0.74799	0.62878	0.8037	0.61025	0.61166
	$Aktp^{308}$ mem	0.73222	0.672	0.7602	0.68092	0.60012	0.87218	1.16506	0.67392	0.73924	0.73222
	Total Akt mem	1.0152	1.1	1.00008571	0.93064286	1.1887	0.9557	1.21135714	1.00725714	1.01635714	1.0152
	PDK1m	0.8586	0.6422	1.0124	0.8586375	0.8586375	0.8585125	0.8586125	0.8585125	0.8585375	0.8586
Peak width	$Aktp^{308}$	2.47747748	2.46846847	2.5045045	1.73873874	2.4954955	2.57657658	2.74774775	3.75675676	2.46846847	2.47747748
	$Aktp^{308}$ mem	0.31818182	0.41363636	0.28636364	0.36590909	0.68636364	0.1	0.75	0.13409091	0.31363636	0.31818182
	Total Akt mem	0.11508951	0.4859335	0.09974425	0.37595908	0.75959079	0.08695652	0.80051151	0.10230179	0.11508951	0.11508951
	PDK1m	0.84615385	0.73076923	1.11538462	0.84615385	0.84615385	0.84615385	0.84615385	0.84615385	0.84615385	0.84615385
SSE (Peak time)		0.11445556	17.2988222	0.14451111	25.5251222	24.2993556	0.74638889	25.4566444	0.67594444	0.11705556	0.11445556
SSE (Peak amplitude)		0.24273908	17.4845627	0.2023636	25.6976773	24.6350033	0.78535348	25.5644043	0.63793369	0.20308284	0.20108573
SSE (Peak width)		3.45455094	17.581469	0.23635653	25.7966449	24.7937962	0.13474699	25.5891491	0.18176186	0.25240084	0.25501408
SSE Score (overall)		3.81174558	20.6531836	3.9007061	27.2833629	27.3361826	5.00112782	28.8669423	10.0199148	3.79146548	3.81174558
Minimum SSE Score:	**3.791465482**										

		M2-1	M2-2	M2-3	M2-4	M2-5	M2-6	M2-7	M2-8	M2-9	M2-10
Peak time	$Aktp^{308}$	1.18	1.18	1.17333333	1.16333333	1.27333333	1.2	1.13	1.15	1.19	1.18
	$Aktp^{308}$ mem	0.23333333	0.23333333	0.41	0.21666667	0.96333333	0.74333333	0.16666667	0.96333333	0.21	0.23666667
	Total Akt mem	0.86	0.86	0.9	0.86	6.06	6.06	0.8	6.08	0.84	0.88
	PDK1m	1.1	1.1	1.15	1.1	1.1	1.1	1.1	1.1	1.1	1.1
Peak amplitude	$Aktp^{308}$	0.63809	0.63809	0.52795	0.69481	0.50637	0.40365	0.76043	0.64722	0.81786	0.6371
	$Aktp^{308}$ mem	0.74094	0.74094	0.66214	0.80588	0.6627	0.60694	0.94682	1.16818	0.7274	0.74272
	Total Akt mem	1.03722857	1.03722857	1.05562857	1.02702857	0.86251429	1.11651429	0.97445714	1.17955714	1.02598571	1.03892857
	PDK1m	1.0193	1.0193	0.8514125	1.1219	1.019275	1.019225	1.0192375	1.0192875	1.0192875	1.0192375
Peak width	$Aktp^{308}$	3	3	3.06306306	2.97297297	1.9009009	2.98198198	3.08108108	3.37837838	4.33333333	2.99099099
	$Aktp^{308}$ mem	0.39772727	0.39772727	0.67727273	0.17727273	0.52954545	0.91590909	0.08636364	1.01590909	0.14772727	0.41590909
	Total Akt mem	0.11253197	0.11253197	0.17391304	0.0971867	0.52685422	1.24808184	0.07928389	1.24296675	0.0971867	0.11508951
	PDK1m	0.92307692	0.92307692	0.73076923	1.23076923	0.92307692	0.92307692	0.92307692	0.92307692	0.92307692	0.92307692
SSE (Peak time)		0.64977778	0.64977778	0.41064444	0.66988889	25.6896556	25.7194778	0.76134444	25.8402444	0.6958	0.63947778
SSE (Peak amplitude)		0.19984939	0.74835663	0.6034312	0.73635205	25.858615	26.0351111	0.80183823	25.9421982	0.69287498	0.73877419
SSE (Peak width)		5.1562491	0.22769093	0.36948058	0.16042351	25.9710419	26.1237295	0.1102219	25.9691382	0.14308574	0.22228941
SSE Score (overall)		6.00587627	6.00587627	5.88808477	6.25413136	27.3291053	30.2463381	6.84185405	31.7474823	13.4628019	5.93344375
Minimum SSE Score:	**5.888084771**										

		M3-1	M3-2	M3-3	M3-4	M3-5	M3-6	M3-7	M3-8	M3-9	M3-10
Peak time	$Aktp^{308}$	0.86333333	0.94	0.77666667	0.86333333	0.96333333	0.69	0.93333333	0.89	0.85333333	0.86333333
	$Aktp^{308}$ mem	0.82333333	0.9	0.74	0.82666667	0.92333333	0.65666667	0.89666667	0.81666667	0.83	0.82333333
	Total Akt mem	0.22	0.24	0.22	0.3	0.24	0.22	0.24	0.22	2.04	0.22
	PDK1m	1.05	1.05	1.05	1.05	1.05	1.05	1.05	1.05	1.05	1.05
Peak amplitude	$Aktp^{308}$	0.90142	0.79265	0.9492	0.90074	0.74127	0.9684	0.92867	0.90764	0.89822	0.90142
	$Aktp^{308}$ mem	1.77394	1.55116	1.87502	1.77258	1.44668	1.91742	1.83808	1.7614	1.78084	1.77394
	Total Akt mem	1.42305714	1.42434286	1.42172857	1.42134286	1.42452857	1.42045714	1.42464286	1.42247143	1.42385714	1.42305714
	PDK1m	0.5944	0.3898125	0.804025	0.59445	0.5944375	0.5944	0.5944	0.5944125	0.5944125	0.5944
Peak width	$Aktp^{308}$	1.97297297	1.74774775	2.27027027	1.96396396	1.71171171	2.51351351	2.79279279	2.26126126	1.89189189	1.97297297
	$Aktp^{308}$ mem	0.43636364	0.39090909	0.48863636	0.43409091	0.38409091	0.52954545	0.61363636	0.425	0.44318182	0.43636364
	Total Akt mem	1.38618926	1.50127877	1.31969309	1.36828645	1.54475703	1.2915601	2.03069054	0.88746803	2.39386189	1.38618926
	PDK1m	0.57692308	0.46153846	0.73076923	0.57692308	0.57692308	0.57692308	0.57692308	0.57692308	0.57692308	0.57692308
SSE (Peak time)		0.66078889	0.5937	0.72837778	0.54122222	0.58732222	0.82487778	0.59522222	0.65661111	1.13451111	0.66078889
SSE (Peak amplitude)		0.95218985	0.63309402	0.68108064	0.53239699	0.65291899	0.72977634	0.59586575	0.65304148	1.12335917	0.65182913
SSE (Peak width)		1.59249858	0.92687137	1.37914064	1.0992324	0.84656424	1.45355802	1.28756606	1.19916033	1.70417027	1.21960114
SSE Score (overall)		3.20547732	2.96420668	3.76264782	3.05406384	2.56012378	4.78488337	6.25222112	3.70091726	5.32610018	3.20547732
Minimum SSE Score:	**2.560123784**										

Table for Text S6. Scores of the peak features for 10 instances of each model (10 members at random from the ensemble) for M1-M3. The ratio with data indicates how closely each model resembles the peak characteristics of the experimental time-series, using 1 to denote a perfect match. The sum of squared error is evaluated using the peak characteristics within each model ensemble.

References

Chen, W.W., et al. (2009) Input-output behavior of ErbB signaling pathways as revealed by a mass action model trained against dynamic data, Mol Syst Biol, 5, 239.
