# Supplementary material for: Non-canonical Activation of Akt in Serum-Stimulated Fibroblasts, Revealed by Comparative Modeling of Pathway Dynamics
Source: PLoS Comput Biol. 2015 Nov 10;11(11):e1004505. doi: 10.1371/journal.pcbi.1004505 (PMC4640559; doi:10.1371/journal.pcbi.1004505)
Supplement: S1 Text — (PDF) [file pcbi.1004505.s008.pdf]

Supplemental Material for "Non-Canonical Activation of Akt in Serum-stimulated Fibroblasts, Revealed by Comparative Modeling of Pathway Dynamics"

Supplemental Text S1. **Construction of the alternative models M1-M5**.

The recruitment effect (in M1-M2) was modeled as an unknown input, which acts as a time-variant multiplier to PIP3's recruitment reaction of Akt to the membrane (#1 of Table 1). In the retention model (M3), the membrane-bound phosphorylated Akt (Aktp308m) is hypothesized to have to "states": $Aktp^{308}mf$ (the normal membrane-localized state of $Aktp^{308}$) and $Aktp^{308}mt$ (the state that cannot dissociate from the membrane). The "retention effect" was implemented using an artificial species called "retention" that converts $Aktp^{308}mf$ to $Aktp^{308}mt$. To balance this effect, we add a spontaneous reversal, in which the membrane-trapped state ($Aktp^{308}mt$) reverts to the normal membrane-localized form ($Aktp^{308}mf$) and restores the ability of $Aktp^{308}$ to dissociate from the membrane. In M4, we encode the general concept of a "dephosphorylation effect" using an artificial "dephosphorylation" species that drives the conversion of the normal phosphatase (Phosphatase) into an inaccessible state (InaccPhosphatase), and using a spontaneous reverse reaction for conversion of the inactive phosphatase into its normal functional phosphatase. Finally in M5 we introduced a hypothesized "Phosphorylation" species that acts in conjunction with PIP3:PDK1m to phosphorylate Akt at the membrane.
